# N^6^-Methyladenosine Modification of ANLN Enhances Hepatocellular Carcinoma Bone Metastasis

**DOI:** 10.7150/ijbs.73570

**Published:** 2023-01-22

**Authors:** Hao Zheng, Zhang-Jun Cheng, Bo Liang, Zhen-Guang Wang, Yuan-Ping Tao, Sheng-Yu Huang, Jun-sheng Ni, Hui-Fen Li, Le Yang, Sheng-Xian Yuan, Jennifer Wu, Takumi Kawaguchi, Hrishikesh Samant, Wei-Ping Zhou, Dai-Min Xiang, Yuan Yang

**Affiliations:** 1 Third Department of Hepatic Surgery, Third Affiliated Hospital, Naval Medical University, Shanghai 200438, China.; 2 Key Laboratory of Signaling Regulation and Targeting Therapy of Liver Cancer (SMMU), Ministry of Education, Shanghai 200438, China.; 3 Department of Organization Sample Bank, Shanghai Key Laboratory of Hepatobiliary Tumor Biology (EHBH), Shanghai 200438, China.; 4 Department of Hepato-Pancreato-Biliary Centers, Zhong Da Hospital, School of Medicine, Southeast University, Nanjing 210009, China.; 5 Department of General Surgery, The Second Affiliated Hospital of Nanchang University, Nanchang, 330000, China.; 6 Department of Hepatobiliary and Pancreatic Surgery, The 10th People's Hospital, Tongji University, Shanghai 200433, China.; 7 Division of Hematology and Medical Oncology, Perlmutter Cancer Center, NYU Langone Health, New York, NY, USA.; 8 Division of Gastroenterology, Department of Medicine, Kurume University School of Medicine, Kurume, Japan.; 9 Hrishikesh Samant, Division of Gastroenterology and Hepatology, LSU Health Science Center, Shreveport, LA, USA.; 10 State Key Laboratory of Oncogenes and Related Genes, Shanghai Cancer Institute, Renji Hospital, Shanghai Jiao Tong University School of Medicine, Shanghai 200032, China.

**Keywords:** hepatocellular carcinoma, bone metastasis, N^6^-methyladenosine, anillin actin binding protein, DZNeP

## Abstract

Bones are categorized as the second most prevalent location of extra-hepatic metastasis in Hepatocellular Carcinoma (HCC), which is linked to an extremely poor prognosis due to limited therapeutic options. N^6^-methyladenosine (m^6^A) is a prominent modification involved in HCC, but the exact mechanisms on how m^6^A modifications induce HCC bone metastases (BM) remain unclear. The key modulators responsible for the abundant m^6^A RNA modification-induced HCC BM was found to be the *METTL3* and *YTHDF1*. The expression of Anillin actin-binding protein (*ANLN*) was dramatically higher in HCC with BM tissues, and its messenger RNA (mRNA) stability was enhanced via m^6^A epitranscriptomic regulation by *METTL3* and *YTHDF1*. High *METTL3* and *YTHDF1* expression along with nuclear *ANLN* protein was clinically correlated with BM in HCC patients. Furthermore, HCC BM was attributed to over-expression of nuclear *ANLN* forming a transcriptional complex with *SP1* which enhanced *KIF2C* transcriptional activity to activate the mTORC1 pathway, therefore increased the expression of *RANKL* and disproportionated *RANKL-OPG* expression in bone microenvironment leading to malignant neoplasms invade bone tissue. In addition, inhibition of *ANLN* m^6^A modification by DZNeP attenuated HCC BM. This data provides meaningful understanding of the modulation and association of m^6^A epitranscriptomic-regulated BM in HCC, and moreover, defines potentially valuable therapeutic targets.

## Background

Many cancer types exhibit preferential metastasis to the bone niche, which contributes significantly to both morbidity and mortality 1. Bone metastasis (BM) has been observed in approximately 38.5 percent of individuals with extra-hepatic metastases in hepatocellular carcinoma (HCC) 2, 3, representing 11.7 percent of HCC patients who get curative resections 4. HCC patients with BM also have intense pain, pathological fractures, and other nerve compression disorders 5, with an average survival time of 7.4 months 6. Hence, gaining a better knowledge of the clinical and genetic processes driving HCC's metastatic growth to the bone is likely to facilitate improvements in patient prognosis and management.

N^6^-methyladenosine (m^6^A) methylation is discovered as the most common and ubiquitous modification in eukaryotic messenger RNAs (mRNAs) 7. In mammalian cells, the m^6^A modification is dynamic and reversible, and it regulates mRNA stability, splicing, transport, localization, and translation, as well as RNA-protein interactions 8. The m^6^A methyltransferases (also known as writers: *METTL3, METTL14,* and *WTAP*) install the m^6^A modifications and m^6^A demethylases (erasers: *FTO* and *ALKBH5*) remove them. Moreover, specific RNA-binding proteins (readers: *YTHDF1/2/3, eIF3, IGF2BP1/2/3,* and *HNRNPA2B1*) directly or indirectly bind to the m^6^A motif to influence RNA function 9. From the perspective of cancer, multiple m^6^A regulators are reportedly dysregulated, functioning as oncogenes or tumor suppressors as per the context 10. Cancer stem cell generation, epithelial-mesenchymal transition (EMT), cancer metabolism, and signal transduction through regulation of mRNA stability or protein translation of downstream effectors have all been linked to dysregulation of m^6^A modifications or m^6^A regulators 11. The importance of m^6^A modification in HCC initiation and progression is being increasingly recognized with collective research efforts starting to clarify the complex functions played by m^6^A modifications and the dysregulation of m^6^A regulators 12, 13. However, detailed investigations focusing on their role in HCC BM are still lacking.

Anillin actin-binding protein (*ANLN*) is a highly conserved actin binding protein playing a crucial part in cell mitosis and functions as a positive regulator of cell division and growth 14. More recently, *ANLN* is found to be increasingly expressed in multiple malignancies and is strongly associated to tumor initiation and progression 15-20 and notably in HCC it is overexpressed and associated with poor prognosis 21. Furthermore, knockdown of ANLN inhibits liver tumorigenesis and HCC growth 20, 22-24. Nevertheless, the role of *ANLN* in HCC BM has remained obscure. The physiological function of m^6^A modification of ANLN in HCC BM was demonstrated in this study, and it was postulated that ANLN could be a new predictive biological marker and therapeutic target for HCC BM.

## Materials and Methods

### Clinical samples

The Institutional Review Board at Third Affiliated Hospital, Naval Medical University (Shanghai, China) approved this study, and it was executed in conformity with the Declaration of Helsinki (as revised in 2013). Before the trial began, the patients signed a written informed consent form. **Supplementary Table 1 and 2** lists the clinical features of each HCC group.

### Cell cultures

The HCC cell lines (Huh-7, HCCLM3, PLC, Hep3B, and Bel-7402) and human umbilical vein endothelial cells (HUVEC) cells and RAW264.7 were purchase from the American Type Culture Collection (ATCC; Manassas, VA, USA) and kept in Dulbecco's modified Eagle's medium (DMEM) supplemented with 10% (v/v) fetal bovine serum (FBS; both Gibco; Thermo Fisher Scientific, Inc., Waltham, MA, USA). Short tandem repeat (STR) sequencing indicated all cell lines were not contaminated by other cells such as HeLa (Biowing Applied Biotechnology Co., Ltd., Shanghai, China). The cells were kept in humidified incubators with 5% CO_2_ at 37 °C. For treatment, indicated HCC cells were stimulated with Wnt3a (2 ng/mL). As per the defined conditions, indicated cells were pre-treated with XAV939 (Wnt inhibitor, 10 μM) and INK-128 (mTORC1 inhibitor, 5 µM). The reagents mentioned above were all obtained from Sigma-Aldrich (St. Louis, MO, USA).

### Animal models

All the mouse experiments which were performed according to the Naval Medical University's approved guidelines. The investigation complied with all applicable ethical regulations related to animal research. Using a 100 µl Hamilton Microliter syringe, all the groups of 1 × 10^7^ luciferase-labeled Huh-7 cells in 50 µl 1 x phosphate-buffered saline (PBS) was injected intracardially in the left ventricle of nu/nu, female 4-6-week-old nude mice 25. After injecting 4.0 mg luciferin (Gold Biotech, St. Louis, MO, USA) in 50 microliters of saline intraperitonially, the IVIS@ Lumina II system (Caliper Life Sciences, Hopkinton, MA, USA) was utilized for monitoring bone metastases for 10 minutes. The organs of the hindlimbs were excised and preserved for histological examination.

### Statistical Analysis

Statistical analysis was performed in collaboration with the bioinformatics department. Each figure and figure legend shows the sample size (n), as well as the statistical test employed and the associated p-values. The data analysis did not exclude any samples or animals. GraphPad Prism Software (GraphPad Software, San Diego, CA, USA) and SPSS version 19.0 were employed for all statistical analyses (SPSS Inc., Chicago, IL, USA). P<0.05 was deemed significant. By using three independent biological repeats, all the quantitative experiments were repeated.

### Other Methods

The material and methods are listed in detail in the Supplementary material.

## Results

### Abnormally increased m^6^A modification is associated with HCC BM

**Figure 1:**
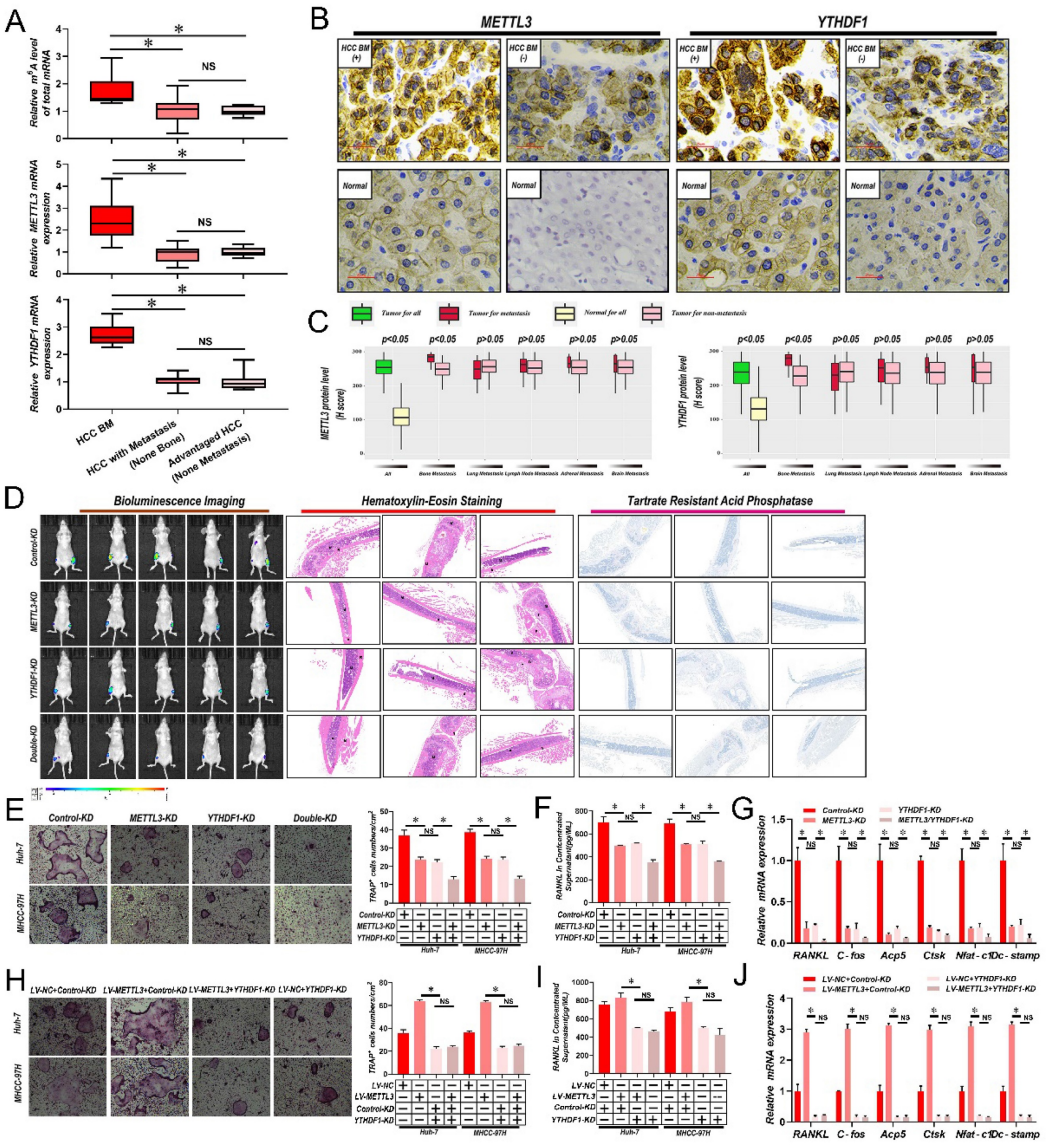
** Abnormally increased *METTL3 and YTHDF1*-induced m^6^A modifications are associated with HCC BM. A.** Total m^6^A levels of total mRNA (Upper panel), *METTL3* (Middle panel) and* YTHDF1*(Down panel) transcript levels in 6 HCC with BM (BM HCC), 13 HCC with metastasis (non-bone), and 10 advanced stage HCC, as assessed using LC-MS/MS and qRT-PCR analysis. **B**. Representative IHC staining of *METTL3* and *YTHDF1* in matched HCC and adjacent normal tissues. Scale bars = 25 µm. **C**. IHC H-Scores from 265 HCC cases comparing the relative staining of *METTL3* and *YTHDF1*. Of these 265 patients, 45 acquired BM, 53 had lung metastasis, 40 developed LNM, 10 suffered from adrenal gland metastasis, and 9 developed brain metastases. Note: In every eligible organ group, multiple organ metastases were included. **D.** Representative BLI images (Left panel), H&E-stained images of bone metastatic loci (Middle panel, scale bars, Magnification × 20), Osteoclast TRAP staining (Right panel, Magnification × 20) from each group (n =5 mice per group). M: marrow; B: bone; T: tumor. Note: H&E-stained and TRAP staining images from the same nude mouse. **E**. Osteoclast differentiation assays were co-cultured with conditioned media from *METTL3/YTHDF1* knockdown and control HCC cells supplemented with M-CSF (50 ng/ml) and RANKL (100 ng/mL), magnification × 200. **F**. Concentration of *RANKL* in the culture medium measured by ELISA from *METTL3/YTHDF1* knockdown and control HCC cell clones. **G**. Osteoclastogenesis differentiation-related factors mRNA level of RAW264.7 cells with conditioned medium from (**G**) was measured by qRT-PCR treated. **H**. The *METTL3* overexpression and control HCC cells were infected with *YTHDF1*-KD or Control-KD. The in vitro osteoclast differentiation assays were performed, magnification × 200. **I**. Concentration of *RANKL* in the culture medium measured by ELISA from *METTL3* overexpression and control HCC cells were infected with *YTHDF1*-KD or Control-KD HCC cell clones. **J**. Osteoclastogenesis differentiation-related factors mRNA level of RAW264.7 cells with conditioned medium from (**J**) was measured by qRT-PCR treated. For(A&C) Unpaired Student's t-test, (E-J) one-way ANOVA. Error bars shows mean ± SD derived from n = 3 independent experiments, *p < 0.05; **p < 0.01; ***p < 0.001, NS p>0.05. m^6^A, N^6^-methyladenosine; HCC BM, hepatocellular carcinoma bone metastasis; eHCC, early-stage HCC; qRT-PCR, quantitative reverse transcription-polymerase chain reaction; LC-MS/MS, liquid chromatography tandem mass spectrometry; IHC, immunohistochemical; mRNA, messenger RNA; HUVECs, human umbilical vein endothelial cells; M-CSDF, macrophage colony-stimulating factor; BLI, bioluminescence imaging; H&E, hematoxylin and eosin; TRAP, tartrate resistant acid phosphatase positive; ANOVA, analysis of variance; SD, Standard Deviation; LNM, lymph node metastasis; NS, not significant.

To determine if m^6^A modified RNAs were associated with BM, firstly m^6^A RNA levels in 6 samples of HCC with bone metastasis (BM), 13 HCC with metastasis (non-bone), and 10 advanced stage HCC without metastasis were compared via liquid chromatography-tandem mass spectrometry (LC-MS/MS). Interestingly, there was a significant increase in the overall m^6^A RNA levels in HCC patient samples with BM in comparison with samples with non-bone metastasis or no metastasis; while no significant differences were recorded between non-bone metastasis and no metastasis HCC cases (**Fig. 1A and Supplementary Table 3**). Then, by employing quantitative reverse transcription polymerase chain reaction (qRT-PCR) analysis, the mRNA levels of significant m^6^A writers (*METTL3, METTL14, KIAA1429*, and *WTAP*), erasers (*ALKBH5* and *FTO*), and readers (*YTHDF1, YTHDF2*, and *YTHDF3*) were compared across all samples. We found that only the core methyltransferase writer *METTL3* and reader *YTHDF1* were considerably increased in HCC patient samples with BM in comparison with samples from non-bone metastasis or without metastasis (**Fig. 1A and Supplementary Table 3**). Again, no significant differences were found between non-bone metastasis and no metastasis cases. Then it was analyzed whether correlations existed between *METTL3* and *YTHDF1* protein expression and metastasis to different organs in 265 HCC cases. Notably, significantly higher expression of METTL3/YTHDF1 was found in HCC tumors with BM compared to HCC tumors without BM. In contrast, no differences were detected between HCC tumors with and without metastasis of the lung, lymph node, adrenal gland, or brain (**Fig. 1B & C**), therefore we focus *METTL3* and *YTHDF1* on HCC bone metastasis.

### *METTL3* and *YTHDF1* depletion impairs HCC BM

Considerably greater levels of *METTL3* and *YTHDF1* were discovered, in cell lines with higher invasive and metastatic abilities, such as MHCC-97H, HCCLM3, and Huh-7, in comparison to cell lines with lesser metastatic potential, such as PLC, Hep3B, and Bel-7402 (**Supplementary Fig. 1A**). Then *METTL3* and *YTHDF1* were knocked down in MHCC-97H and Huh-7 HCC cells (**Supplementary Fig. 1B and C**). As shown in **Fig. 1D**, lower luciferase signals were detected in nude mice inoculated with *METTL3/YTHDF1-KD* Huh-7 cells compared with mice bearing control Huh-7 cells. Histological staining of tumor sections with hematoxylin and eosin (H&E) showed that the tumor lesion area in mouse limbs was greatly reduced by knockdown of *METTL3* and *YTHDF1* along with reduced tartrate resistant acid phosphatase (TRAP), indicative of clear decreases in osteoclast activity. Since osteoclastogenesis is recognized as essential for the establishment of BM, it was next examined how *METTL3/YTHDF1* expression in HCC contributes to osteoclast differentiation. Indeed, fewer TRAP^+^ multinucleated cells were observed when bone marrow cells (BMCs) were cultured with conditioned medium from *METTL3/YTHDF1*-KD HCC cells compared with control cells (**Fig. 1E**). Moreover, the levels of *RANKL* which promote osteoclastogenesis decreased, while *OPG* which is a decoy receptor for *RANKL* that prevents osteoclastogenesis increased in conditioned medium after *METTL3/YTHDF1* knockdown (**Fig. 1F & Supplementary Fig. 1D**). Additionally, culturing RAW246.7 cells with HCC-conditioned medium from METTL3/YTHDF1-KD HCC cells revealed significantly decreased expression of osteoclast differentiation/activation markers including *RNAKL, C-fos, Acp5, Ctsk, Nfat-c1*, and *Dc-stamp* (**Fig. 1G and Supplementary Fig. 1E**). Following, overexpression of *METTL3* enhanced osteoclast differentiation; however; knockdown of *YTHDF1* impaired these effects (**Supplementary Fig. 1F-I and Fig. 1H-J**). Lastly, supporting these findings, investigation of HCC in The Cancer Genome Atlas (TCGA) database using gene expression profiling interactive analysis (GEPIA) indicated that the expression of *METTL3* and *YTHDF1* were positively associated with several known factors related to osteoclastogenesis (**Supplementary Fig. 1J**). Collectively, these data suggested that the *METTL3* and *YTHDF1* function to promote HCC BM.

### Hypermethylated m^6^A correlates with oncogene over-expression in HCC BM

**Figure 2:**
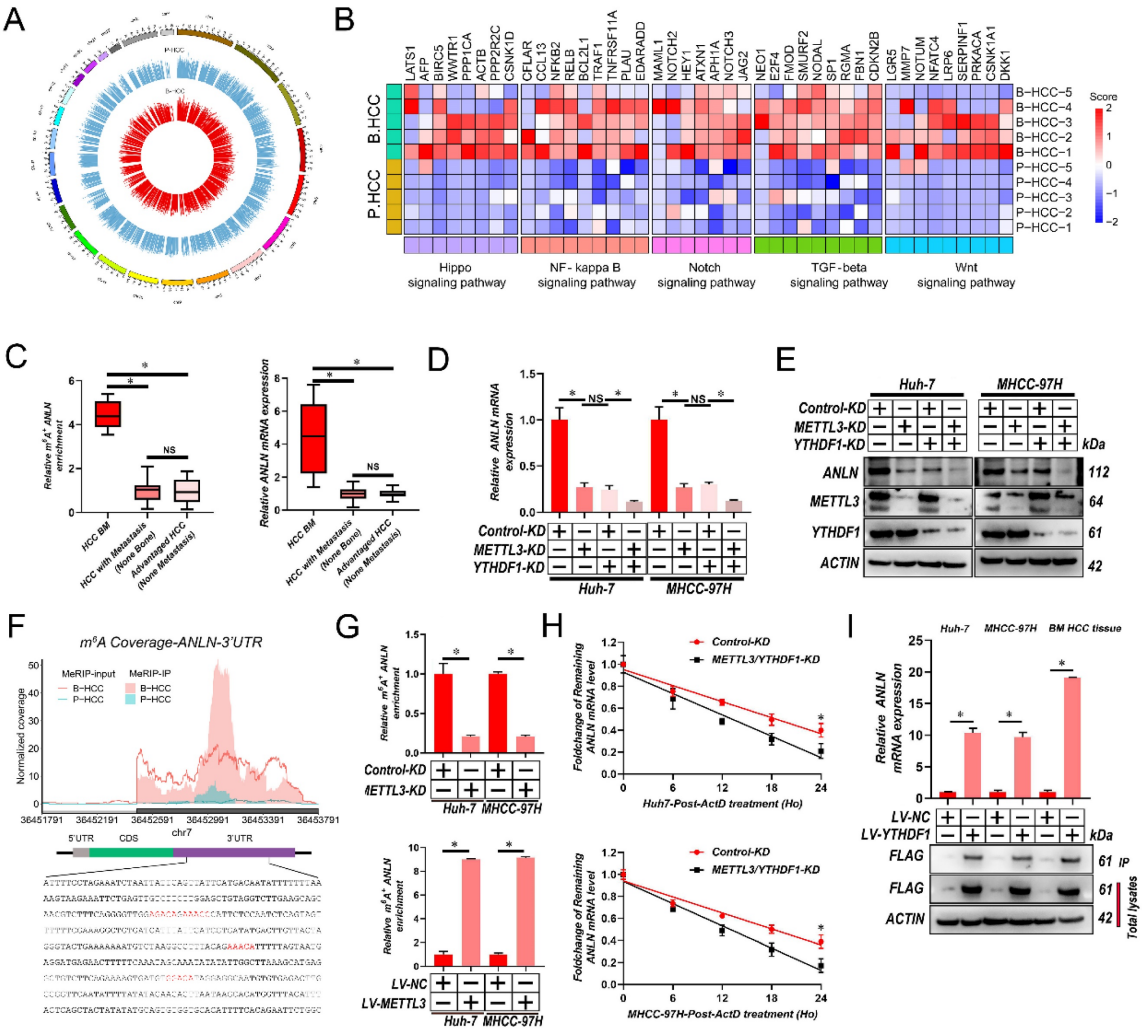
***METTL3* and* YTHDF1* induces m^6^A modification of ANLN 3′-UTR to enhance its mRNA stability in HCC BM. A**. Circos plot demonstrating m^6^A peaks on human chromosomes. The human genome's chromosome map is presented as the circos plot's outermost layer. Red or green bars (5 B-HCC vs. 5 P-HCC) indicate increased or decreased m^6^A peaks, respectively. All target m^6^A found by sequencing is shown by the second outermost circle. **B**. Heatmap showing the m^6^A hypermethylation tendency in B-HCC for 35 representative genes involved in key cancer-related pathways. The hypermethylated m^6^A sites were detected using the m^6^A-seq data for 5 B-HCC and 5 P-HCC. m^6^A-seq was performed once for each sample. Colored from blue to red to indicate low to high H-score values, respectively. **C**. *ANLN* m^6^A and transcript levels in 6 HCC with BM (BM HCC), 13 HCC with metastasis (non-bone), and 10 advanced stage HCC, as assessed using MeRIP-PCR and qRT-PCR analysis. **D**. qRT-PCR analysis of *ANLN* mRNA in Huh-7 and MHCC-97H cells after knockdown *METTL3* or/and *YTHDF1*. **E**. The ANLN protein levels in Huh-7 and MHCC-97H cells infected with *METTL3/YTHDF1-KD* measured by western blotting. **F**. Coverage plots of m^6^A peaks in ANLN gene comparing matched B-HCC (n=5 independent biological samples) versus P-HCC (n=5 independent biological samples) by m^6^A-seq. Plotted coverages are the median of the n replicates presented. Eight DRACH motif sites were identified in ANLN 3′-UTR. The inset presents bp 242-696-bp of ANLN 3′-UTR region, with the DRACH motif highlighted by red text. **G**. *METTL3*-mediated *ANLN* m^6^A modifications were demonstrated using MeRIP-qPCR analysis. The m^6^A modification of *ANLN* was enhanced when *METTL3* was up-regulated, but it was reduced when *METTL3* was knocked out. **H.** qRT-PCR analysis of ANLN mRNA in Huh-7 and MHCC-97H cells infected with/without *METTL3/YTHDF1-KD* following Actinomycin D treatment for 0-24 h. **I**. ANLN mRNA was quantified by qRT-PCR as the percentage of input and graphed as fold enrichment relative to control. Immunoblot analysis of FLAG-YTHDF1 in the input and IP is shown on bottom panels. For (G, I) Paired Student's t-test, (C&D) one-way ANOVA, (H) Repeated-measures analysis of variance analysis. Error bars indicate mean ± SD were derived from n = 3 independent experiments, *p < 0.05; **p < 0.01; ***p < 0.001, NS p > 0.05. m^6^A, N^6^-methyladenosine; HCC BM, hepatocellular carcinoma bone metastasis; UTR, untranslated region; B-HCC, HCC BM focus; P-HCC, HCC primary focus; qRT-PCR, quantitative reverse transcription-polymerase chain reaction; mRNA, messenger RNA; MeRIP-qPCR, methylated RNA immunoprecipitation-qPCR; ANOVA, analysis of variance; SD, Standard Deviation

To map the transcriptome-wide m^6^A modifications in HCC BM, m^6^A-seq was performed for 5 HCC primary focus (P-HCC) and 5 HCC BM focus (B-HCC) tissues. We found that the majority of the m^6^A sites (approximately 35%) were present in 3′ untranslated region (UTR) (**Supplementary Fig. 2A**), identifying 16,968-32,626 m^6^A sites in 8,618-12,309 mRNAs for each sample (**Supplementary Fig. 2B**). The m^6^A sites were enriched within the coding and 3'UTR sequence, and intriguingly, referentially accumulated in the CDS regions immediately downstream of translation initiation sites (**Supplementary Fig. 2C & D**). Consistent with previous reports 26, a proportion of the m^6^A sites conformed to the mammalian DRACH consensus motif (**Supplementary Fig. 2E**). Further analysis of our m^6^A-seq data revealed 6,681 differentially regulated m^6^A sites between P-HCC focus and B-HCC focus tissues (**Fig. 2A**). Among these, 5,994 m^6^A sites in 4,430 mRNAs were hypermethylated and 687 m^6^A sites in 547 mRNAs were hypomethylated in BM focus versus primary focus tissues (**Supplementary Fig. 2F**), indicating that m^6^A sites are frequently hypermethylated in HCC BM. The frequent m^6^A hypermethylation in BM HCC led us to categorize the function of the m^6^A-hypermethylated mRNAs. As shown in **Fig. 2B** and **Supplementary Fig. 2G,** the hypermethylated mRNAs were mainly enriched in several cancer-related pathways including the Wnt, Hippo, Notch, transforming growth factor-β (TGF-β), and nuclear factor-κß (NF-κß) pathway. Together, these data indicate that m^6^A hypermethylation is a frequent event in BM HCC and that the hypermethylated mRNAs are enriched in known oncogenic pathways.

To better establish the association between m^6^A modifications and gene expression in HCC BM, we performed RNA sequencing (RNA-Seq) on the same cohort of 5 P-HCC and 5 B-HCC tissues. Analysis of these data identified 1,665 up- and 1,317 down-regulated mRNAs in B-HCC compared to P-HCC tissues (**Supplementary Fig. 2H**). Intersecting the m^6^A-Seq and RNA-Seq further revealed that expression and methylation of 68 mRNAs (up-regulated: Fold-Change>16, p-value<0.0001; and hypermethylated: Fold-Change>10, p-value<0.05) were positively correlated (**Supplementary Table 4**). Further interrogation of these 68 genes via MeRIP-qPCR and qRT-PCR assays in cohort (**Fig. 1A**) highlighted *ANLN* displayed strong correlations between the extent of m^6^A modification, mRNA and HCC BM (**Fig. 2C and Supplementary Table 5**). These data suggest that the m^6^A RNA modification enhances the mRNA expression of *ANLN* associated with HCC BM.

### METTL3 and YTHDF1 Induces m^6^A Modification of ANLN 3'-UTR to Enhance its mRNA stability

Next, we validated the mRNA and protein levels *ANLN* were reduced by *METTL3/YTHDF1* knockdown in HCC cells (**Fig. 2D and E**). Our m^6^A-seq results revealed that 4 DRACH motifs and m^6^A peaks within the 244-800-bp region of the 3′-UTR of *ANLN* were selectively increased in HCC BM focus tissues (**Fig. 2F**). In further analyses, MeRIP-qPCR assays were utilized for validating the m^6^A modification of *ANLN* mRNA in HCC cells and for confirming the function of *METTL3* in the m^6^A modification of *ANLN* mRNA (**Fig. 2G**). Taking into consideration the positive regulation of mRNA levels of *ANLN* by m^6^A modification, it was investigated then, if the m^6^A modification influences the stability of *ANLN* mRNA. The HCC cells were treated with actinomycin D, an inhibitor of transcription, for the indicated times. As shown in **Fig. 2H**, knockdown of *METTL3* and *YTHDF1* reduced the half-life of *ANLN* mRNA in HCC cells. Moreover, in HCC cells and B-HCC tissue, an RNA immunoprecipitation (RIP) assay suggests that YTHDF1 recognizes and binds with the *ANLN* mRNA (**Fig. 2I**). Notably, examination of *ANLN* in HCC using GEPIA database indicated *ANLN* expression was significantly associated with the *METTL3* and *YTHDF1* expression (**Supplementary Fig. 2I**). Collectively the findings suggest that *ANLN* expression is maintained by *METTL3*-mediated m^6^A modification via YTHDF1-dependent *ANLN* mRNA stability.

### High *METTL3, YTHDF1* expression and nuclear *ANLN* levels are associated with poor survival and BM in HCC

*ANLN* is a highly dynamic protein, with notable variations in its expression and localization during various phases of the cell cycle 27. Consequently, the nuclear and cytoplasmic distribution of *ANLN* in 265 HCC cases by immunohistochemical (IHC) analysis was evaluated, respectively. As shown in **Supplementary Fig. 3A and B**, both the nuclear and cytoplasmic staining of *ANLN* were increased in 265 HCC tissues in comparison to the neighboring normal tissues. Whether correlations existed between *ANLN* nuclear/cytoplasmic staining and metastasis to different organs was then analyzed. As expected, higher nuclear *ANLN* protein expression was found in HCC tumors with BM compared to HCC tumors without BM although no correlations existed with metastases to other sites including lung, lymph node, adrenal gland, and brain. Moreover, higher cytoplasmic stain for *ANLN* was not correlated with metastasis to different organs including bone, lung, lymph node, adrenal gland, and brain (**Supplementary Fig. 3B**). Furthermore, Kaplan-Meier survival curves presented that the HCC cases with elevated *METTL3, YTHDF1,* and nuclear *ANLN* expression had poorer overall survival (OS) and recurrence-free survival (RFS), respectively (**Supplementary Fig. 3C-E**). In contrast, cytoplasm *ANLN* staining exhibited no correlation with survival in 265 HCC cases (**Supplementary Fig. 3F**). Altogether, these outcomes suggest that a direct correlation of elevated *METTL3, YTHDF1,* and nucleus *ANLN* expression is associated with BM and low survival probability in HCC.

### The *METTL3* and *YTHDF1*-*ANLN* axis promotes HCC BM

**Figure 3:**
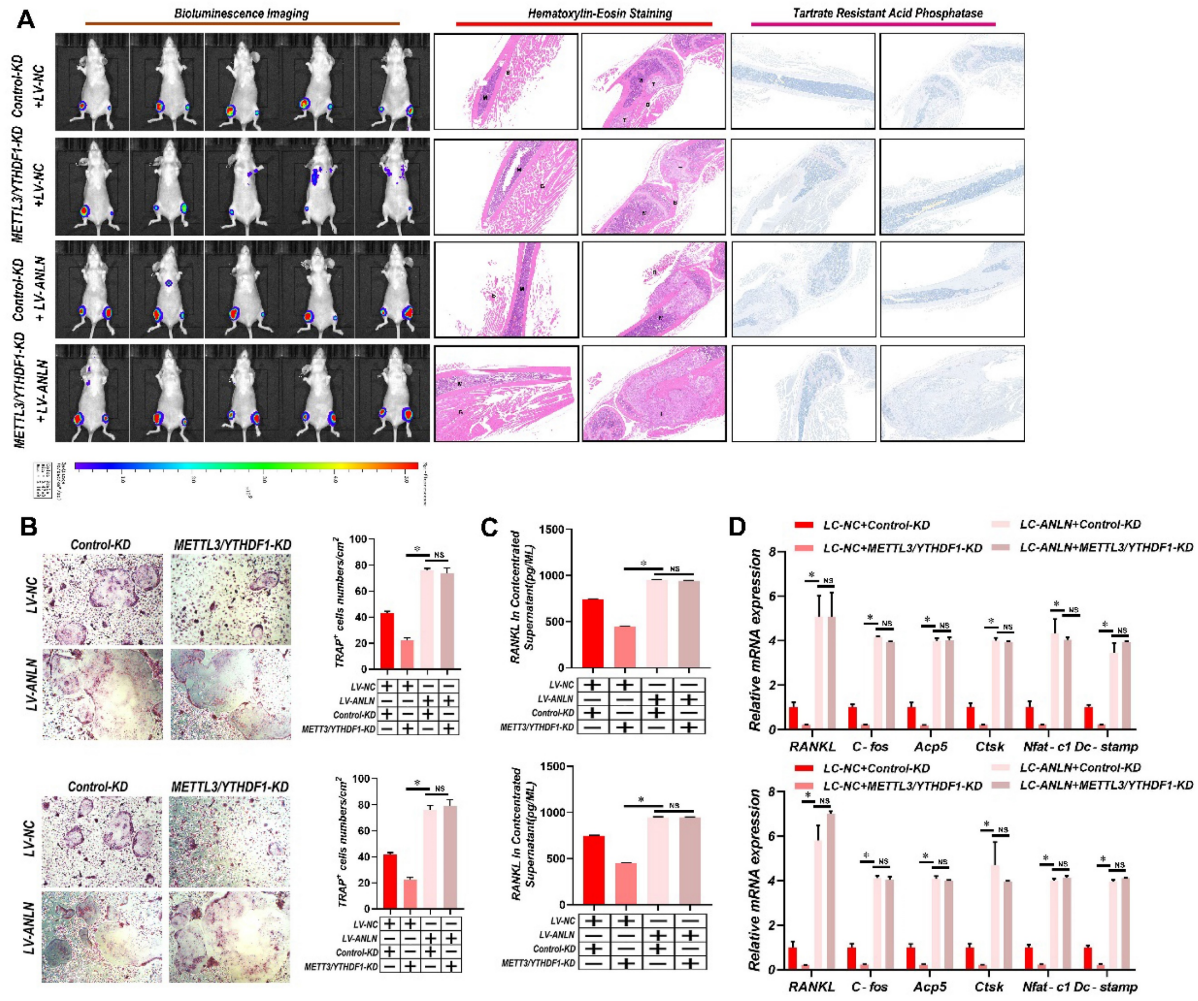
***METTL3* and *YTHDF1* -ANLN Axis Promotes HCC BM. A.** Representative BLI images (Left panel), H&E-stained images of BM loci (Middle panel, Magnification × 20), Osteoclast TRAP staining (Right panel, Magnification ×20) from each group (n = 5 mice per group). M: marrow; B: bone; T: tumor. Note: H&E-stained and TRAP staining images from the same nude mouse. **B.** The *METTL3/YTHDF1*-KD and control HCC cells were infected with LV-NC or LV-*ANLN*. The in vitro migration osteoclast differentiation assays were performed, magnification × 200. **C**. Concentration of *RANKL* in the culture medium measured by ELISA from *METTL3/YTHDF1*-KD and control HCC cells clones. **D**. Osteoclastogenesis differentiation-related factors mRNA level of RAW264.7 cells with conditioned medium from (**B**) was measured by qRT-PCR treated. For (**B**-**D**) one-way ANOVA. Error bars indicate mean ± SD were derived from n = 3 independent experiments, *p < 0.05; **p < 0.01; ***p < 0.001, NS p > 0.05. HCC BM, hepatocellular carcinoma bone metastasis; BLI, bioluminescence imaging; H&E, hematoxylin and eosin; TRAP, tartrate resistant acid phosphatase positive; ANOVA, analysis of variance; SD, Standard Deviation; NS, not significant.

To learn more about *ANLN*'s oncogenic function in HCC, further comparisons were made between *ANLN* knockdown and overexpression HCC cells (**Supplementary Fig. 4A-D**). Notably, knockdown of *ANLN* markedly suppressed HCC BM *in vivo* and osteoclast differentiation activities (**Supplementary Fig. 4E & F and Supplementary Fig. 4H-J**). Conversely, ectopic *ANLN* expression significantly enhanced cell osteoclast differentiation (**Supplementary Fig. 4G and Supplementary Fig. 4K-M**). Furthermore, the reintroduction of *ANLN* in *METTL3/YTHDF1* knockdown HCC cells restored BM capabilities *in vivo* (**Fig. 3A**) along with osteoclast differentiation (**Fig. 3B-D**). Taken together, these findings indicated that *METTL3* and *YTHDF1* promote HCC BM through the upregulation of *ANLN* expression.

### Interactions between *ANLN* and *SP1* are indispensable for *SP1*-mediated oncogenic transcription

**Figure 4:**
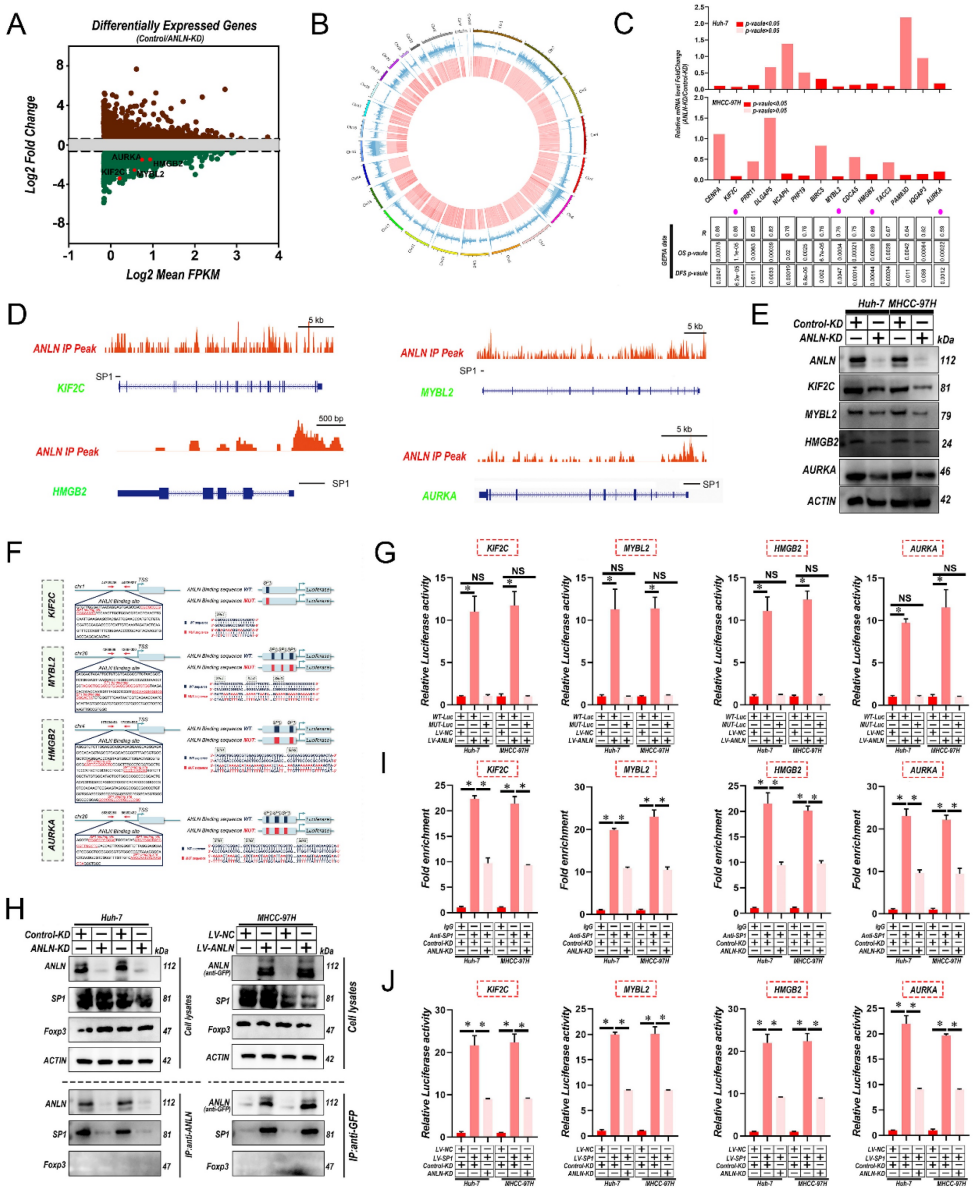
** Interaction between *ANLN* and* SP1* is indispensable for *SP1*-mediated Oncogenic transcription. A.** MA plot showing the mean log2expression against the log2-fold change of the RNA-seq data obtained from control and *ANLN*-KD Huh-7 cell lines. Green and brown depicts decreased and increased gene expression in the *ANLN*-KD group, respectively. **B.** Circos plot showing *ANLN*-binding peaks on human chromosomes. The outermost layer of the circos plot is a chromosome map of the human genome. The second outermost circle represents all target *ANLN*-binding peaks detected by sequencing. **C.** qRT-PCR confirmed that the mRNA level of 14 genes with the highest correlation with *ANLN* in HCC (base on GEPIA database) with *ANLN*-KD. The data in the grid below are R (coefficient of correlation, Pearson test); DFS value (disease-free survival, log-rank test); OS value (Overall survival, the log-rank test). **D**. Genome browser screenshot *ANLN* peaks (Red, based on CUT-Tag seq) at the *KIF2C, MYBL2, HMGB2* and *AURKA* gene prompter loci, respectively. The thick black line is the predicted binding site of *SP1* (Transcription Factor *SP1* ChIP-seq Clusters from ENCODE 3 Source data version: ENCODE 3 Nov 2018). **E**. Western blot analysis of the level of *KIF2C, MYBL2, HMGB2*, and *AURKA* protein level in *ANLN* knockdown and control HCC cells. **F**. Schematic representation of the* ANLN* binding region of *KIF2C, MYBL2, HMGB2,* and *AURKA*, respectively. Luciferase reporter plasmids that included the WT or MUT (*ANLN* binding site sequence of *KIF2C, MYBL2, HMGB2, and AURKA*) were constructed. **G**. Luciferase activity in stable HCC cell clones transfected with luciferase reporters containing WT-*ANLN*-binding site or MUT-*ANLN*-binding site. Data are presented as the relative ratio of firefly luciferase activity to Renilla luciferase activity. **H.** The indicated HCC cell lysates were prepared and immunoprecipitated (IP) with anti-GFP (for GFP-tagged *ANLN*), or anti-*ANLN* (for endogenous *ANLN*) antibodies. IP and cell lysates were analyzed by western blotting. Foxp3 served as negative control. **I**. HCC cells (control-KD or *ANLN*-KD) were collected and subjected to ChIP assay using anti-SP1 antibody, followed by qPCR analysis. Graphs show ChIP-qPCR measurement of *SP*1 enrichment at the *KIF2C, MYBL2, HMGB2, and AURKA* promoter regions. **J**. Luciferase activity in stable HCC cell clones co-transfected with luciferase reporters containing WT-*ANLN*-binding site and *ANLN*-KD. Data are presented as the relative ratio of firefly luciferase activity to Renilla luciferase activity. For (C) Paired Student's t-test, (G, I and J) one-way ANOVA. Error bars indicate mean ± SD were derived from n = 3 independent experiments, *p < 0.05; **p < 0.01; ***p < 0.001, NS p > 0.05. HCC BM, hepatocellular carcinoma bone metastasis; BLI, bioluminescence imaging; qRT-PCR, quantitative reverse transcription-polymerase chain reaction; ChIP, chromatin immunoprecipitation; SEM, standard error of the mean; ANOVA, analysis of variance; SD, Standard Deviation; NS, not significant.

To further identify the potential substrates of *ANLN*, firstly RNA sequencing was utilized for comparing the expression profiles between Huh-7-control and ANLN-KD cells. Among *ANLN*-regulated genes, total 578 genes were greatly up-regulated, and 1,012 genes were down-regulated in Huh-7-ANLN-KD cells (**Fig. 4A**). As mentioned above, the nuclear localization of *ANLN* was associated with HCC BM. Combined with *ANLN* as an essential regulator of nucleoli biogenesis 28, a Cleavage Under Targets and Tagmentation (CUT&Tag) assessment on *ANLN* was conducted to specify its targets. Global mapping analysis demonstrated that the reads were most distributed amongst promoter regions (~39.23%) (**Fig. 4B and Supplementary Fig. 5A & B**), implying that ANLN may have a new role in regulating gene transcription in HCC. Combinatorial analysis of CUT&Tag versus RNA-seq data revealed that 238 of the differentially up-regulated genes were regulated by *ANLN* and exhibited *ANLN* binding at their respective promoters (**Supplementary Fig. 5C**). Thereafter, 14 genes showing upregulated expression levels in HCC (fold-change>2, p-value <0.01) and positive correlations with *ANLN* (r > 0.55, p-value <0.05) in the GEPIA database were chosen for further study (**Fig. 4C & D, Supplementary Fig. 5D & E and Supplementary Table 6 & 7**). Subsequent qRT-PCR analysis confirmed that 4 genes: *KIF2C, MYBL2, HMGB2,* and *AURKA,* were all downregulated after knockdown of *ANLN* in HCC cells (**Fig. 4C, upper panel**). Western blot analysis revealed their protein levels were also downregulated (**Fig. 4E**). Consistently, IHC analysis of the 265 HCC cases suggested that protein levels of *KIF2C, MYBL2, HMGB2,* and *AURKA* were positively correlated with the levels of nuclear but not cytoplasmic ANLN levels (**Supplementary Fig. 5F & G and Supplementary Table 8**). Instructively, activation of the *KIF2C, MYBL2, HMGB2,* and *AURKA* promoters was enhanced by *ANLN* overexpression in HCC cells (**Fig. 4F & G**) while chromatin immunoprecipitation (ChIP)-PCR assays further confirmed the direct binding of *ANLN* to their respective promoter regions (**Supplementary Fig. 5H**). It was recently reported that *ANLN* binds to particular transcription factors (TFs) in the nuclear context 29 so the potential functional collaborations between ANLN and TFs in HCC was investigated. Immunoprecipitation-mass spectrometry (IP-MS) analysis identified that ANLN bound to the well-known transcription factor *SP1*, and this interaction was further confirmed in co-immunoprecipitation (co-IP) assays (**Fig. 4H**). Consistently, interrogation of the JASPAR database (http://jaspar.genereg.net/) indicated that several *SP1* binding sites were located within the ANLN-CUT-Tag peak of the *KIF2C*, *MYBL2*, *HMGB2*, and *AURKA* promoters (**Fig. 4D & F**). In addition, the mutation of *SP1* binding site within the *KIF2C, MYBL2, HMGB2,* and *AURKA* promoter regions diminished the distinct activation differences in the *KIF2C, MYBL2*, *HMGB2*, and *AURKA* promoters between *ANLN* overexpression and control cells (**Fig. 4G**). Consistently, the ability of *SP1* to bind to the promoter regions of *KIF12C, MYBL2, HMGB2,* and *AURKA* was significantly reduced after *ANLN* knockdown (**Fig. 4I & J** and **Supplementary Fig. 5I**). Lastly, analysis of HCC samples in the GEPIA database demonstrated that *SP1* was positively correlated with *KIF2C, MYBL2, HMGB2,* and *AURKA* in HCC (**Supplementary Fig. 5J**). These results confirm that nuclear ANLN protein cooperatively combines with *SP1* to enhance *KIF2C, MYBL2, HMGB2,* and *AURKA* expression in HCC.

### *ANLN* facilitates HCC BM through *KIF2C/mTORC1* signaling

Previous studies have indicated that *KIF2C* enhanced mammalian target of rapamycin complex1 (mTORC1) signal transduction in HCC 30, while recent studies have reported that activation of mTORC1 signaling promotes osteoclast formation 31, 32.

**Figure 5:**
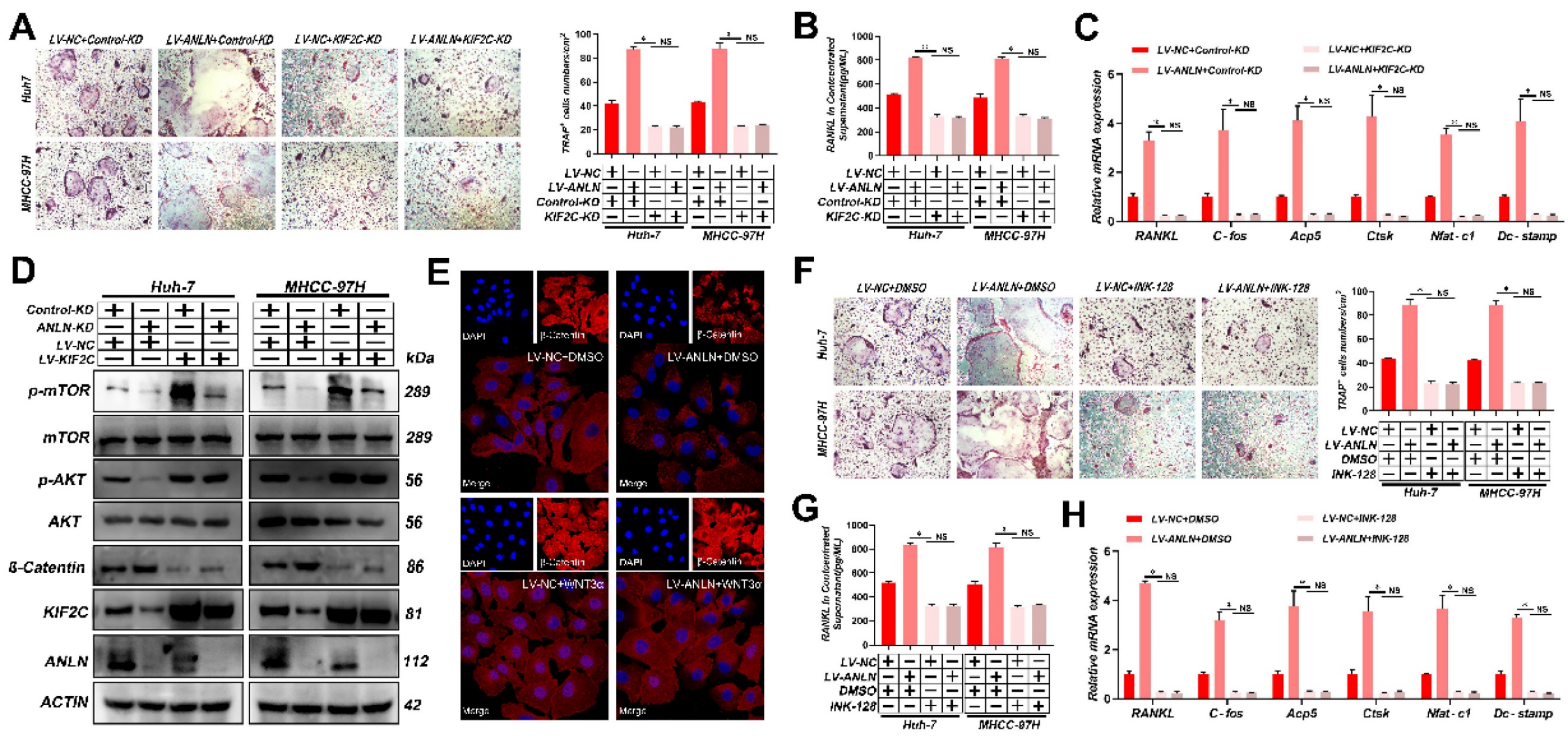
***ANLN* Facilitates HCC BM Through *KIF2C/mTORC1* Signaling. A**. Osteoclast differentiation assays were co-cultured with conditioned media from *ANLN* overexpression and control HCC cells were infected with *KIF2C* knockdown group supplemented with M-CSF (25 ng/mL) and RANKL (100 ng/ml), magnification × 200. **B**. Concentration of *RANKL* in the culture medium measured by ELISA from (**A**). **C**. Osteoclastogenesis differentiation-related factors mRNA level of RAW264.7 cells with conditioned medium from (**A**) was measured by qRT-PCR treated. **D.** Western blot analysis of the level of *ANLN, KIF2C,* and the phosphorylation of mTOR protein in indicated treated cells. **E**. Immunofluorescence staining for nuclear β-catenin after applying the LV-NC or LV-*ANLN* in MHCC-97H cells with or without Wnt3a treatment (2 ng/mL) for 8 h. β-Catenin is labelled in red. **F**. *ANLN* overexpression and control HCC cells were treated with or without INK-128, in vitro osteoclast differentiation assays supplemented with M-CSF (25 ng/mL) and RANKL (100 ng/ml), magnification × 200. **G**. Concentration of *RANKL* in the culture medium measured by ELISA from (**F**). **H**. Osteoclastogenesis differentiation-related factors mRNA level of RAW264.7 cells with conditioned medium from (**F**) was measured by qRT-PCR treated. **I.** Representative BLI images (Left panel), H&E-stained images of bone metastatic loci (Middle panel, scale bars, 500 µm), Osteoclast TRAP staining (Right panel, scale bars, 500 µm) from each group (n =3 mice per group). M: marrow; B: bone; T: tumor. **J.** Western blot analysis of the level of indicated protein level in BM-HCC (n=4) and no-BM-HCC (n=4) group. For (A-C, F-H) one-way ANOVA. Error bars indicate mean ± SD were derived from n = 3 independent experiments, *p < 0.05; **p < 0.01; ***p < 0.001, NS p>0.05. HCC BM, hepatocellular carcinoma bone metastasis; mRNA, messenger RNA; qRT-PCR, quantitative reverse transcription-polymerase chain reaction; HUVEC, human umbilical vein endothelial cell; BLI, bioluminescence imaging; TRAP, tartrate resistant acid phosphatase positive; H&E, hematoxylin and eosin; ANOVA, analysis of variance; SEM, standard error of the mean; SD, Standard Deviation; NS, not significant.

Notably, knockdown of *KIF2C* in HCC cells significantly reduced osteoclast differentiation activity promoted by ectopic *ANLN* expression (**Fig. 5A-C** and **Supplementary Fig. 6A-E**). Thus, it was further explored whether the *ANLN-KIF2C* axis enhances HCC BM via mTORC1 signaling. Indeed, mTOR phosphorylation was downregulated in *ANLN*-KD HCC cells and this could be partially recovered by ectopic *KIF2C* (**Fig. 5D**), indicating that the *ANLN-KIF2C* axis likely activates mTORC1 signaling. Activation of mTORC1 promotes osteoclast formation by regulating *RANKL/OPG* expression via activating the Akt pathway and negatively regulating β-catenin 31, 32. Consistently, decreased phosphorylation of AKT and increased β-catenin were observed in *ANLN*-KD HCC cells that could be partially recovered by ectopic expression *KIF2C* (**Fig. 5D**). Moreover, subcellular fraction and immunofluorescence analysis indicated that overexpression of *ANLN* impaired the nuclear accumulation of β-catenin (**Fig. 5E** and **Supplementary Fig. 6F**). Furthermore, the specific mTORC1 inhibitor INK-128 abolished the osteoclast differentiation ability differences measured between *ANLN* overexpression and control HCC cells (**Fig. 5F-H** and **Supplementary Fig. 6G & H**). Importantly, *KIF2C* depletion or INK-128 treatment both abrogated the ANLN-enhanced BM of HCC cells *in vivo* (**Fig. 5I**). Lastly, analysis of patient-derived HCC tissues indicated that *ANLN, KIF2C* and mTORC1 pathway-related genes were increased in BM-HCC tissues compared to non-BM-HCC tissues, while *p-AKT* and *β-*catenin pathway related genes were decreased (**Fig. 5J**). Together, these outcomes suggest that the *KIF2C/mTORC1* pathway is required for *ANLN*-enhanced HCC BM.

### Suppression of *ANLN* m^6^A modification attenuates HCC BM

**Figure 6:**
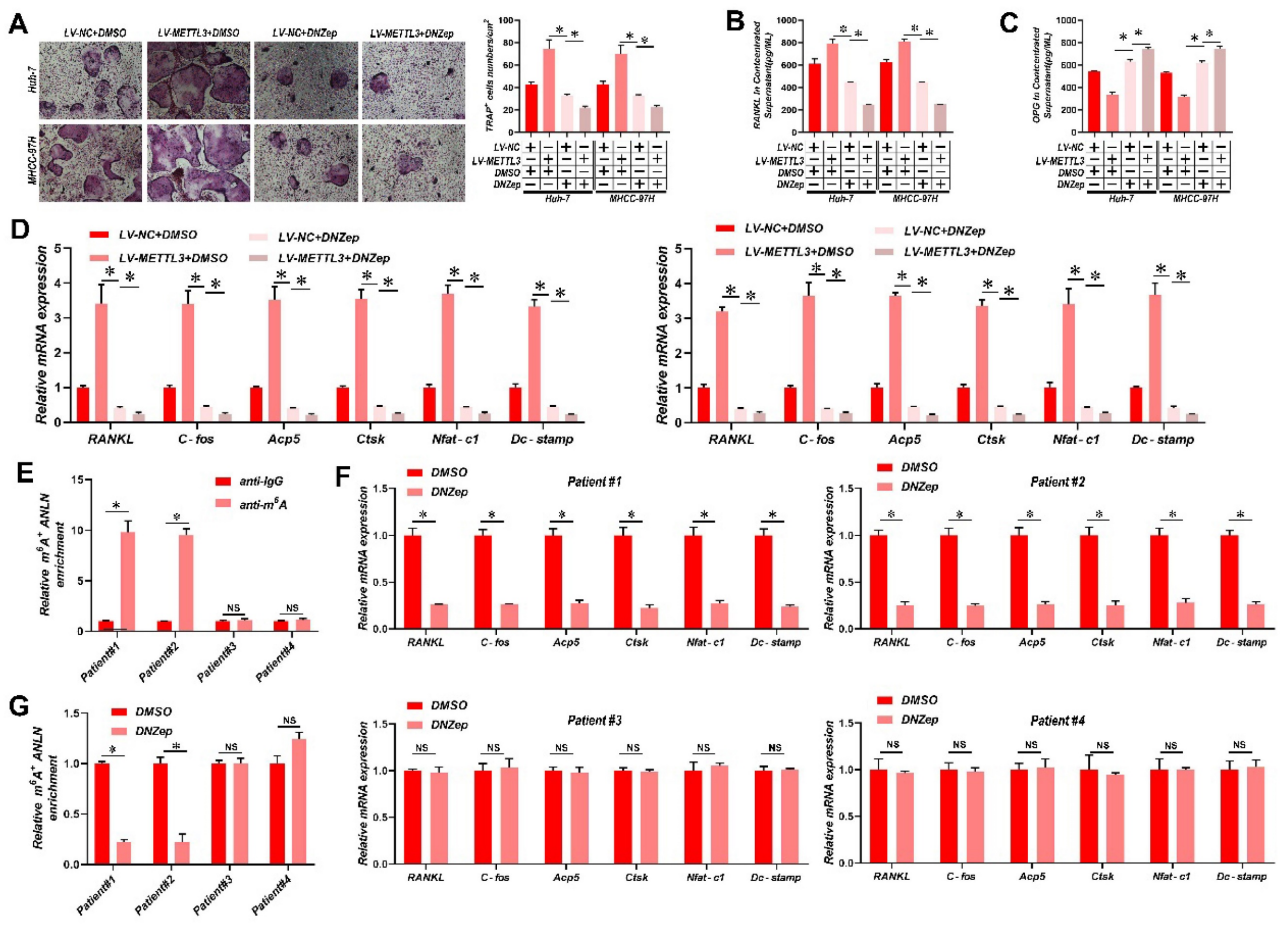
** Suppression of *ANLN* m^6^A modification attenuates HCC BM. A.** Osteoclast differentiation assays were co-cultured with conditioned media from Huh-7 and MHCC-97H cell lines infected with LV-*METTL3* or LV-NC were treated with vehicle (DMSO) or DNZep (10nM) supplemented with M-CSF (25 ng/mL) and RANKL (100 ng/ml), magnification × 200. **B&C.** Concentration of *RANKL* (**B**) and *OPG* (**C**) in the culture medium measured by ELISA from Huh-7 and MHCC-97H cell lines infected with LV-*METTL3* or LV-NC were treated with vehicle (DMSO) or DNZep (10nM). **D.** Osteoclastogenesis differentiation-related factors mRNA level of RAW264.7 cells with conditioned medium from (**A**) was measured by qRT-PCR treated. **E.** MeRIP-qPCR analysis was employed to assessment the *ANLN* m^6^A modifications in the HCC tissues from patient#1-4. **F.** MeRIP-qPCR analysis was employed to assessment the *ANLN* m^6^A modifications from PDX model treated with Vehicle or DZNep (8 mg/kg per mouse). **G.** Osteoclastogenesis differentiation-related factors mRNA level was measured by qRT-PCR from PDX model treated with Vehicle or DZNep (8 mg/kg per mouse). For (**A** - **D**) one-way ANOVA, (**E** - **G**) Paired Student's t-test. Error bars indicate mean ± SD were derived from n = 3 independent experiments, *p < 0.05; **p < 0.01; ***p < 0.001, NS p > 0.05. m^6^A, N^6^-methyladenosine; HCC BM, hepatocellular carcinoma bone metastasis; BLI, bioluminescence imaging; H&E, hematoxylin and eosin; qRT-PCR, quantitative reverse transcription-polymerase chain reaction; MeRIP-qPCR, methylated RNA immunoprecipitation-qPCR; mRNA, messenger RNA; TRAP, tartrate resistant acid phosphatase positive; DMSO, dimethylsulfoxide; PDX, patient-derived xenograft ANOVA, analysis of variance; SD, Standard Deviation; NS, not significant.

It has been reported that 3-deazaneplanocin A (DZNeP) inhibits RNA methylation 33. DZNeP significantly reduced overall mRNA m^6^A methylation and the m^6^A modification of ANLN mRNA in HCC cells, as revealed in **Supplementary Fig. 7A & B**. Moreover, both the ANLN mRNA and protein levels were down-regulated in DZNeP-treated HCC cells (**Supplementary Fig. 7C & D**). In addition, DZNeP treatment abolished m^6^A hypermethylated *ANLN* and increased *ANLN* mRNA levels in HCC cells with LV-*METTL3* (**Supplementary Fig. 7E & F**), also diminishing the differences in osteoclast differentiation and BM *in vivo* between control and *METTL3*-overexpressing HCC cells (**Fig. 6A-E**). A further observation worth noting is that DNZep treatment also inhibited HCC proliferation (**Supplementary Fig. 7G**). HCC patient-derived tumor xenograft (PDX) models were also established from four HCC patient tissues, two with high and two with low *ANLN* m^6^A modifications, respectively, and employed these to assess the clinical potential of using DZNeP to target m^6^A modified *ANLN* (**Fig. 6F**). These results showed that DZNeP treatment significantly suppressed m^6^A modified *ANLN* mRNA levels and also mRNAs of genes associated with osteoclast differentiation (**Fig. 6G and H**). Furthermore, DZNeP treatment significantly inhibited the tumor growth of the high *ANLN* m^6^A-modified HCC PDX models, while these effects were not evident in the two low *ANLN* m^6^A-modified HCC PDX lines (**Supplementary Fig. 7H**). Assessing the *in vivo* cytotoxicity of these treatments, the vital organs were collected after DZNep treatment but found no obvious side effects (**Supplementary Fig. 7I**). Altogether these outcomes revealed that antagonizing increased levels of m^6^A modified ANLN in HCC significantly inhibits BM.

## Discussion

**Figure 7:**
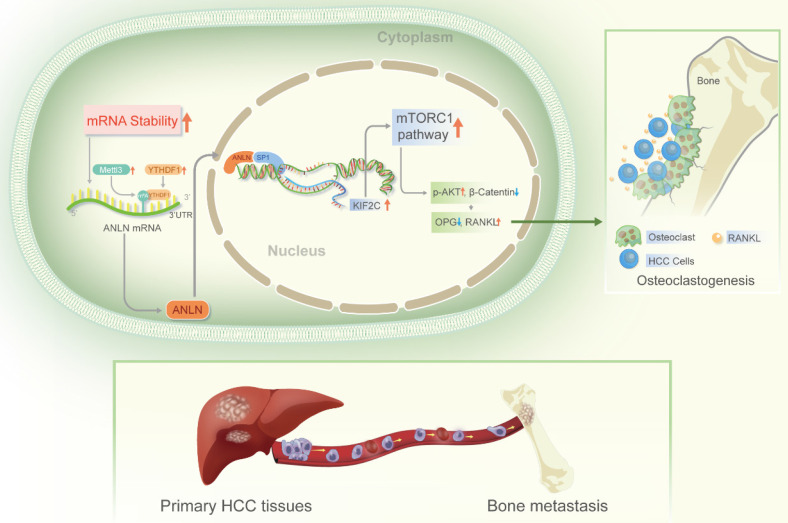
Schematic illustration of the *METTL3* and *YTHDF1* enhanced *ANLN/KIF2C/mTRORC1* axis in driving HCC BM. **Abbreviations:** HCC BM, hepatocellular carcinoma bone metastasis.

In advanced malignancies, BM is a common occurrence, and once established, patient prognosis is extremely poor 34. However, the understanding of molecular mechanisms in BM from HCC is limited. The m^6^A is the most common RNA modification in both mRNA and non-coding RNA 9, and is involved in various human malignant tumors, including HCC 13. According to Wen et al. 35 Long non-coding RNA NEAT1 increases prostate cancer bone metastases via N6-methyladenosine. Nevertheless, links between m^6^A RNA modifications and HCC BM are currently understudied. Here, the m^6^A levels of mRNA were demonstrated to be greatly increased in HCC BM tissues because of the upregulation of the methyltransferase *METTL3* and reader *YTHDF1.* Mechanistically, it was deduced that upregulation of *METTL3* stimulates m^6^A modified ANLN mRNA levels, with *YTHDF1* directly binding to m^6^A sites in ANLN mRNA to maintain ANLN mRNA stability. Nuclear ANLN/SP1 complexes to the *KIF2C* promoters serve to promote the development of HCC BM through activating mTORC1 pathways (**Fig. 7**).

A growing body of evidence suggests that dysregulation of m^6^A modification is linked to tumor metastasis 11. Among the m^6^A modulators, the m^6^A writer *METTL3* or reader *YTHDF1* have been reported to either facilitate or impair carcinogenesis in different cancer types 36. However, detailed investigations about their role in site-specific metastases to bone, especially in HCC, remains unclear. In the present research, it was found for the first time that *METTL3* and *YTHDF1* induced increased m^6^A modifications associated with HCC BM. Furthermore, *METTL3* and *YTHDF1* overexpression in HCC cells were required for the BM process, thus highlighting *METTL3* and *YTHDF1* as potential predictive biomarkers and therapeutic targets for HCC. By employing RNA-seq and m^6^A-seq, promising findings were discovered related to *ANLN* being a significant downstream target of *METTL3* and *YTHDF1* associated with HCC BM. Earlier research has revealed that ANLN exhibits an important part in a variety of cancer related mechanisms, which includes cancer initiation, growth, angiogenesis, and metastasis 14. In HCC it was reported that *ANLN* promotes hepatic carcinogenesis as well as cancer cell proliferation 20, 22-24. Regardless of these reported results, the function of *ANLN* in HCC BM remains unclear. *ANLN* expression and localization have been shown to be dynamic 27 and *ANLN* is primarily nuclear in cancer cells, with only a weak cytoplasmic staining observed, as reported by most of the published clinical investigations 14. Consistently high nuclear expression of *ANLN* was found in HCC, also finding this distribution acts as an important prognostic factor for poor survival. Instructively, the increased nuclear expression of *ANLN* was shown to be associated with HCC BM, for the first time. While the activities of nuclear *ANLN* in tumorigenesis have been unexpectedly overlooked, the nuclear localization of *ANLN* in many cancer cells has been thoroughly described. Through CUT & Tag and RNA-seq, *KIF2C* was identified as a key target gene of ANLN. Furthermore, IP-MS analysis showed that nucleus ANLN and SP1 enhance *KIF2C* transcriptional activity via formation of transcription complex to enhance HCC BM via the mTORC1 pathway. The identification of this functional mechanism enriches the understanding of how HCC BM is promoted while also providing a new direction for the clinical diagnosis and treatment of HCC.

Bone metastasis is a complicated process involving the interaction among the tumor cells and the bone milieu. Elucidating the constitution of the bone milieu and how it interacts with tumor cells assists in understanding the potential mechanism of metastatic organ tendency. The bone microenvironment of RANKL/OPG ratio is a key in regulating osteoclast formation and bone resorption 37,38. Increasing of *RANKL* and/or decreasing of *OPG* are sufficient to encourage the generation of osteoclasts and trigger the bone dissolution process 38. *RANK* is one of the surface receptors of tumor necrosis factor (TNF) family 38. This receptor modulates calcium metabolism and is required for the development, activation, and functionality of osteoclasts 37. Recent research has found that *RANK* is expressed in tumor cells, insinuating its role in tumor metastasis 37. The level of formation of osteoclasts is largely determined by the balance of *RANKL* and *OPG* activities, with elevated levels of *OPG* leading to a reduction in bone resorption 37. *OPG* is the soluble receptor of *RANKL* and belongs to the small integrin binding ligand N-linked glycoprotein family 37,38. *OPG* competes with *RANK* for *RANKL* in this manner, affecting osteoclast generation and ensuing bone resorption by preventing *RANKL-RANK* communication on the osteoclast membrane. Mammalian rapamycin target (mTOR) is considered to be a key cellular factor regulating most basic cellular functions 39. In addition, mTOR has a crucial function in determining intraosseous homeostasis. It is reported that mTOR in osteoclasts regulates osteoclast differentiation, while rapamycin mTOR signal in osteoclasts can eliminate osteoclasts through *RANKL/OPG* pathway 39. These results showed that important participants of m^6^A modifications (*METTL3* and *YTHDF1*) enhance the stability of *ANLN* mRNA through m^6^A epitope transcriptional regulation, in turn with nuclear ANLN forming a transcriptional complex with SP1, which enhances the transcriptional activity of *KIF2C*, and activates the mTORC1 pathway. This increases the expression of *RANKL* and breaks the balance of *RANKL-OPG* expression in bone microenvironment leading to malignant neoplasms invade bone tissue. This study explored the relationship between m^6^A methylation and the microenvironment of hepatocellular carcinoma, enriched the understanding of the role of abnormal m^6^A methylation in promoting bone metastasis of hepatocellular carcinoma, and provided new ideas for the clinical diagnosis and treatment of bone metastasis of advanced hepatocellular carcinoma.

The bone marrow has a diverse composition of immune cells and may provide an immune-privileged habitat for disseminated tumor cells 40. Bone metastasis involves many different immune cells 41 and takes place in an individual immunological environment. It is thought that m6A methylation influences the control of the tumor immune milieu, impacting the metastatic processes of a variety of malignancies 41. m6A regulates the differentiation of T cells, the stabilization of Tregs, the maturation of Dendritic Cells (DCs), the polarization of macrophages and the functional modulation of myeloid-derived suppressor cells (MDSCs) 41. m6A modulators are thus involved in tumor immunity and relevant to immunotherapy. Abnormal expression of m6A regulators has the potential to influence anticancer immune functions and modulate tumor metastasis 42. The current research focused on the relationship between the m6A-ANLN axis and the equilibrium of RANKL-OPG expression in the bone microenvironment, immune microenvironment in bone metastasis not be accommodated and should be investigated in greater depth in the future.

Currently, effective therapeutic targets for HCC patients with BM are still not available 34. Therefore, identifying novel therapeutic targets towards improving for HCC patient survival remains an unmet need. The *ex vivo* analysis of patient tissues revealed that BM HCC cases with high m^6^A modifications of *ANLN* mRNA, highlighting the potential therapeutic value of targeting the m^6^A modification in *ANLN* in HCC. Indeed, these findings using the RNA methylation inhibitor DZNeP 33, showed that DZNeP inhibits *ANLN* m^6^A methylation and effectively attenuates the malignant phenotype and BM *in vivo* of HCC cells exhibiting *ANLN* overexpression. The correlation between *ANLN* m^6^A methylation levels and DZNeP responses using PDX models was further validated. The results showed that PDXs with high m^6^A *ANLN* methylation level displayed significant growth inhibition upon DZNeP treatment, while PDXs with low m^6^A *ANLN* methylation level expression did not respond to DZNeP, implying that employing DZNeP to inhibit the m^6^A-ANLN axis may be a promising therapeutic option for HCC BM patients.

## Conclusion

In summary, this work uncovered the functions of the m^6^A-*ANLN* axis induced by *METTL3*/*YTHDF1* in HCC BM, and provided compelling evidence that multiple phases of the HCC BM cascade require m^6^A-*ANLN* upregulation. Furthermore, these findings provide understanding of the modulation and linkage of the m^6^A epitranscriptome in regulating BM in the HCC-specific context, also proposing new therapeutic targets for bone metastasis. Finally, the limitations of this study must be acknowledged. The sample size of the cohort needs to be increased, especially the patient subgroups with specific organ metastases. Future studies would benefit from expanding sample sizes in a multicenter environment to establish a clearer relationship between the expression of *METTL3, YTHDF1, ANLN* and bone metastasis. In addition, as with all experimental models, this *in vivo* bone metastasis model may not fully reflect the disease in patients, so further verification with other models would be beneficial to substantiate both the phenotype and molecular mechanisms of the m6A-METTL3/YTHDF1-ANLN axis in driving HCC BM.

## Supplementary Material

Supplementary materials and methods, figures and tables.

## Abbreviations

m^6^A: N6-methyladenosine
HCC: hepatocellular carcinoma
BM: bone metastasis
ANLN: Anillin actin-binding protein
HCC: early advantage HCC
IHC: immunohistochemical
TMAs: tissue microarrays
OS: overall survival
RFS: recurrence-free survival
MIH: metastasis-inclined HCC
MAH: metastasis-averse HCC
qRT-PCR: quantitative reverse transcription PCR
UTR: untranslated region
mTOR: mammalian target of rapamycin
SP1: specificity protein 1
mRNA: messenger RNA
TSS: transcription start site
WTAP: Wilm's tumor 1-associating protein
FTO: fat mass and obesity-associated protein
LC-MS/MS: liquid chromatography-tandem mass spectrometry
EMT: epithelial-mesenchymal transition
RANKL: receptor activator of NF-κB ligand
OPG: osteoprotegerin
IP-MIS: immunoprecipitation-mass spectrometry
