# Supplementary material for: N6-Methyladenosine Modification of ANLN Enhances Hepatocellular Carcinoma Bone Metastasis
Source: Int J Biol Sci. 2023 Jan 22;19(4):1009–23. doi: 10.7150/ijbs.73570 (PMC10008695; doi:10.7150/ijbs.73570)
Supplement: Supplementary file 1 — Supplementary materials and methods, figures and tables. [file ijbsv19p1009s1.pdf]

## Supplementary Materials and Methods

### Clinical samples

For the RNA N6-methyladenosine ($m^6A$)-seq and RNA-seq analyses, five pairs of HCC BM and primary tumor samples were obtained from HCC patients who had undergone hepatic resection at the Eastern Hepatobiliary Surgery Hospital (EHBH). 6 samples of HCC with BM (BM HCC), 13 of HCC with metastasis (non-bone), and 10 advanced-stage HCC samples who had undergone hepatic resection at the same hospital were analyzed by liquid chromatography-tandem mass spectrometry (LC-MS/MS), methylated RNA immunoprecipitation-quantitative reverse transcription-polymerase chain reaction (MeRIP-qPCR), and qRT-PCR (**Supplementary Table 1**). Samples were immediately frozen in liquid nitrogen. The 10 samples (5 HCC primary focus and 5 HCC BM focus tissues) were used for further verification of the RNA $m^6A$-seq data by MeRIP-qPCR analysis. Immunochemistry (IHC) was used to analyze 265 paired HCC and normal specimens from consecutive patients who had undergone hepatic resection at the EHBH; the clinical characteristics of this cohort are tabulated in **Supplementary Table 2**. These specimens were immediately frozen in liquid nitrogen, followed by formalin fixation, paraffin embedding, and pathological verification.

### Ultra-high performance liquid chromatography-multiple-reaction monitoring-tandem mass spectrometry

Oligo dT magnetic beads were used to purify mRNA. Two hundred nanograms of mRNA were treated with nuclease P1 (0.5 U, Sigma-Aldrich, St. Louis, MO, USA) in a 25 μL of 10 mM $NH_4OAc$ (pH = 5.3) for 1 h at 42 ℃ after which 3 μL of 1 M $NH_4HCO_3$ and 1 μL of 1 U/μL alkaline phosphatase (Sigma) were added with incubation for 2 h at 37 ℃. The solution was neutralized with 1 μl of 3 M HCl, made up to 50 μL and passed through a 0.22 μm filter (Millipore, Burlington, MA, USA). The samples (10 μl for each injection) were analyzed on a reverse-phase ultra-performance liquid chromatography (UHPLC) C18 column (Agilent Technologies, Santa Clara, CA, USA) with an Agilent 6410 QQQ triple-quadrupole LC mass spectrometer using a positive electrospray ionization mode and quantification using retention times and ion

mass transitions of 268.0 to 136.0 (A) and 282.1 to 150.0 ($m^6A$), with calculations relative to standards run in the same batches. Calibration curves were used to calculate the $m^6A$:A ratio.

**IHC analysis**

IHC was conducted according to a previously published protocol [1]. The sections were heated, followed by incubation with primary antibodies for 30 min and secondary antibodies for 1 h at room temperature (RT). Details of the antibodies are shown in **Supplementary Table 9**. The sections were then counterstained with hematoxylin, developed with diaminobenzidine, and examined by three blinded pathologists. The H-scores [2] for the staining in selected fields at 400x magnification for *METTL3*, *YTHDF1*, cytoplasmic *ANLN*, *KIF2C*, *MYBL2*, *HMGB2*, and *AURKA* were determined; staining intensities were scored as 0, 1, 2, or 3 for negative, weak, intermediate, and strong brown staining, respectively. Total cell numbers and numbers of stained cells in each field were counted and the average percentages of positive cells were determined. The H-score was determined as: (% of cells stained at intensity category 1 × 1) + (% of cells stained at intensity category 2 × 2) + (% of cells stained at intensity category 3 × 3). The H-scores ranged from 0-300, with 0 indicating negative staining in all cells and 300 representing 100% of cells showing strong staining. Median H-score values were applied as cut-offs to separate low- and high-expression groups. For nuclear *ANLN* staining, the percentage of cells showing moderate-to-strong nuclear staining was determined and the median percentage was used to separate low- and high-expression groups.

**Stable Cell Line Construction**

The *METTL3, ANLN*, *SP1, HMGB2,* and *KIF2C* expressing lentivirus vector *(LV-METTL3-GFP-PRO, termed as LV-METL3; LV-ANLN-GFP-PURO,* termed as *LV-METTL3; LV-METTL3-PURO, termed as LV-METTL3; LV-ANLN; LV-SP1-GFP-PURO*, termed as *LV-SP1*; *LV-HMGB2-GFP-PURO*, termed as *LV-HMGB2; and LV-KIF2C-GFP-PURO,* termed as *LV-KIF2C*) and their control vector (LV-GFP-PURO,

termed as LV-NC) were supplied by Shanghai Yuan zhi Biotechnology Co., Ltd. The *YTHDF1* expressing lentivirus vector (*LV-YTHDF1-FLAG-PURO, termed as LV-YTHDF1*) and its control vector (LV-FLAG-PURO, termed as LV-NC) were supplied by Shanghai Yuan zhi Biotechnology Co., Ltd. Lentiviruses knock down *METTL3 (#1, #2, #3, termed as METTL3-KD1, METTL3-KD2, METTL3-KD3); YTHDF1 (#1, #2, #3, termed as YTHDF1-KD1, YTHDF2-KD2, YTHDF3-KD3); ANLN (#1, #2, #3, termed as ANLN-KD1, ANLN-KD2, ANLN-KD3); KIF2C (#1, #2, #3, termed as KIF2C-KD1, KIF2C-KD2, KIF2C-KD3); MYBL2 (#1, #2, #3, termed as MYBL2-KD1, MYBL2-KD2, MYBL2-KD3); HMGB2(#1, #2, #3, termed as HMGB2-KD1, HMGB2-KD2, HMGB2-KD3); AURKA (#1, #2, #3, termed as AURKA-KD1, AURKA-KD2, AURKA-KD3)* or NC (termed as Negative control) were purchased from Shanghai Yuan zhi Biotechnology Co., Ltd. Cells were plated at $1\times10^5$ cells/well and were transfected with lentivirus, following the provided directions. Puromycin (3 μg/mL; Biosharp, Gunagzhou, China) and/or amplicillin (3 μg/ml; Biosharp) were used to select infected cells for at least one week, and the efficiency of transfection was assessed by qRT-PCR or western blotting. All the targeted sequences are shown in **Supplementary Table 10**.

**RNA extraction, reverse transcription, and qRT-PCR**

Total RNA was extracted from the frozen samples using TRIzol (Takara, Dalian, China), following the provided directions. The RNA quality was examined using a Nanodrop 2000 spectrophotometer and agarose gel electrophoresis. One to two micrograms of the RNA were used for reverse transcription to cDNA using random primers and M-MLV Reverse Transcriptase (Invitrogen, Waltham. MA, USA). RT-PCR was conducted by the Taqman assay or SYBR Green system in a Step One Plus system (Applied Biosystems, Waltham, MA, USA), the relative mRNA levels were determined according to the ΔΔCt values. The sequences of the primers are provided in **Supplementary Table 11**.

**MeRIP-qPCR**

Methylated RNA immunoprecipitation-qPCR was performed using an adaptation of a published method [3]. After denaturation of poly-A-purified RNA for 10 min at 70 °C, it was immediately cooled on ice and treated with an anti-$m^6A$ antibody in 1 mL 50 mM Tris-HCl with 400 U of RNasin Plus RNase Inhibitor (Promega, Madison, WI, USA), 750 mM NaCl, and 0.5% (v/v) Igepal CA-630 (Sigma-Aldrich) for 2 h at 4 ℃. The solution was then incubated with washed Dynabeads Protein G (Invitrogen) for 2 h at 4 °C with gentle shaking. Two elutions of the $m^6A$ RNA were performed with 6.7 mM $N^6$-methyladenosine 5′-monophosphate sodium salt at 4 °C, and the material was precipitated 5 μg glycogen and a one-tenth volume of 3 M sodium acetate in 2.5 volumes of 100% ethanol at −80 °C overnight. $m^6A$ concentrations in the precipitate were measured by qPCR. Fragmented mRNA was probed directly with the anti-$m^6A$ antibody and assessed in a similar manner.

**Enzyme-linked immunosorbent assay**

The levels of *RANK*L and *OPG* in culture supernatants obtained over 48 h were measured using the Human *RANKL* and *OPG* kits (both Ray Biotech), following the provided directions.

**Osteoclast differentiation assays**

Bone marrow cells (BMCs) were obtained from the femurs of four-week-old mice and grown in minimum essential medium (MEM) with 15% FBS and macrophage colony-stimulating factor (M-CSF) (50 ng/mL) for three days. Adherent cells were harvested, washed three times with PBS, and plated in 12-well plates with conditioned medium from treated HCC cells, *M-CSF* (25 ng/mL), and *RANKL* (100 ng/mL) for three days. The cells were stained using a tartrate-resistant acid phosphatase positive (TRAP) kit (Sigma Aldrich), according to directions, to analyze osteoclast and preosteoclast formation. TRAP-stained cells with a minimum of three nuclei were defined as osteoclasts. A minimum of nine fields per plate were evaluated and the mean osteoclast numbers were recorded.

**CCK8 (Cell Counting Kit-8) assay**

A CCK-8 kit (C0038, Beyotime Biotechnology, Shanghai) was used to measure proliferation, as described [4].

**Animal models**

All the mouse experiments which were performed according to the Naval Medical University's approved guidelines. The investigation complied with all applicable ethical regulations related to animal research. To investigate the influence of *ANLN* on HCC cell lines' BM ability, luciferase-labelled Huh7 ($1 \times 10^{7)}$ cells in 50 μL 1× PBS were injected into the left ventricles of four- to six-week-old female nude using a 100-μl Hamilton Microliter syringe as described previously [5]. The BMs were monitored by the IVIS@ Lumina II system (Caliper Life Sciences, Hopkinton, MA, USA) for 10 min after intraperitoneal injection of 4.0 mg of luciferin (Gold Biotech, St. Louis, MO, USA) in 50 μL of saline. The hindlimb organs were removed and fixed for histological examination. The investigators were not blinded.

**RNA immunoprecipitation (RIP) assays**

RIP assays were performed using a Magna RIP RNA-Binding Protein Immunoprecipitation Kit (17–700, Millipore), according to provided directions. Magnetic beads were coated with 5 μg of the appropriate antibodies and incubated with cell lysates overnight at 4 °C. After six washes, the complexed material was treated with proteinase K digestion buffer, followed by extraction of the RNA with phenol-chloroform. Interactions between *YTHDF1* and *ANLN* mRNA were assessed qPCR and expressed relative to the input. Details of the antibodies are provided in **Supplementary Table 9**.

**Chromatin Immunoprecipitation (ChIP)**

The Magna ChIP G Kit (17-611; Millipore) was used in accordance with the manufacturer's directions. After cross-linking of cells with 37% formaldehyde (Sigma F8775) solution (final concentration 1%) for 10 min and quenching with glycine, the PBS-washed cells were resuspended in lysis buffer with protease inhibitors and vortexed intermittently for 15 min at 4 °C. After centrifugation (800 g; 5 min), the

pelleted cells were resuspended in nuclei lysis buffer and sonicated with a Misonix S-4000 sonicator at 50% amplitude 15-s pulse with 90-s rest between each pulse (10 cycles). The size of the chromatin was assessed by electrophoresis and was immunoprecipitated with either 3 μg anti-TCF4 or 3 μg anti-IgG (Millipore 17-10109) overnight at 4 °C. ChIP fragments were examined using RT-PCR and the primers provided in **Supplementary Table 11**.

**Luciferase activity report assay**

Wild-type (WT) and mutant (Mut) *ANLN* bind peaks (shown in **Fig.5H**) were inserted into pGL3 luciferase reporter by BgIII digestion. Huh7 and MHCC-97H cells were seeded in 48-well plates and the adherent cells were co-transfected with pGL3-binding peak-WT and-Mut plasmids together with Renilla using Lipofectamine™ 2000 (Invitrogen) for 48 h. Firefly and Renilla activities were measured and firefly activity was normalized to that of Renilla.

**Immunoprecipitation (IP) and LC-MS**

The Dynabeads Co-Immunoprecipitation Kit (14321D, Thermo Fisher) was used for IP, following the provided directions. Proteins were separated on SDS-PAGE and the bands were digested with trypsin. The peptides were applied to an LC gradient for 2 h and were analyzed on a Q Exactive HF mass spectrometer (Thermo Fisher Scientific). The data were compared with the mouse database by MaxQuant (https://maxquant.org/). The intensity-based absolute-protein-quantification (iBAQ) intensity was used for inter-sample comparisons. The antibodies used are shown in **Supplementary Table 9**.

**Western blotting (WB)**

Cells were lysed in RIPA buffer with protease and phosphatase inhibitors, as well as DTT and benzonase (Sigma-Aldrich). Protein concentrations were measured using a BCA kit (Thermo Scientific Pierce, Loughborough, UK). WB was performed as previously described using NuPAGE@Novex 4-12% Bis-Tris protein gels (Novex, Life Technologies, Carlsbad, CA, USA). The antibodies used are shown in **Supplementary Table 9**.

**Nuclear and cytoplasmic protein extraction**

Cells were fractionated using a cytoplasmic and nuclear protein/RNA extraction kit (Thermo Fisher Scientific) according to the supplied protocol. The loading controls for the nuclear and cytoplasmic fractions were laminB1 and α-tubulin, respectively.

**Immunofluorescence**

Cells ($4x10^4$) were grown in confocal plates, fixed with 4% formaldehyde for 20 min, and permeabilized with 0.1% Triton X-100 for 20 min. The cells were blocked with 5% BSA in Tris-buffered saline with Tween 20 TBST) and were probed with the appropriate primary antibodies overnight at 4 °C. After incubation with secondary antibodies (30 min, RT) and nuclear staining with DAPI, the cells were examined and imaged with a confocal microscope (Olympus, Tokyo, Japan). The antibodies used are shown in **Supplementary Table 9**.

**Patient-Derived Xenografts**

For the patient-derived tumor xenograft (PDX) model, fresh tumor tissues were cut into 3x3 cm sections and implanted subcutaneously into the mouse flanks. The mice were given 3-deazaneplanocin A (DZNeP; 8 mg/kg per mouse) or dimethylsulfoxide (DMSO) orally 7 times for 2 weeks. The growth of the tumors was assessed at the indicated times. This procedure was approved by the EHBH Ethics Committee. Growth rates were calculated as previously described [6].

**DZNeP treatment**

Cells were treated with 10 μM DZNeP (Selleck, Houston, TX, USA; S7120) to prevent $m^6A$ modification. Mice received subcutaneous injections of DZNeP (8 mg/kg) every other day.

**$m^6A$-Seq**

The $m^6A$-Seq method was used to measure $m^6A$ RNA methylation. The library was constructed using the Next® Ultra™ II RNA Library Prep Kit (E7775; NEB), quantified with a Qubit Fluorometer (Thermo Fisher Scientific), and size distributions confirmed with Tape Station D1000 Screen Tape (Agilent Technologies). Sequencing

was done on an Illumina platform using PE150 (Shanghai OE Biotech, Shanghai, China).

**Data processing and peak calling for m$^6$A-seq**

We used Fast p v0.20.0 (https://github.com/OpenGene/fastp) was used to remove adapter and low-quality reads. Both MeRIP and input reads were aligned to the human genome (GRCh38.p12) using HISAT2 v2.1.0 (http://daehwankimlab.github.io/hisat2/) with parameter --rna-strandness RF --fr. The m6A peak calling was performed using the R package MeTDiff v 1.1.0. The P-value < 0.05 was set as the threshold for a significant m6A peak. We used MEME-ChIP v5.0.5 (https://meme-suite.org/meme/tools/meme-chip) to identify enriched motifs in the m6A peak region. The m$^6$A peak distribution on the metagene was plotted by R package Guitar v1.1.1.18. Analysis of differential m6A methylation was also performed using MeTDiff. Peak annotation was performed using ChIPseeker v1.12.1 (https://www.bioconductor.org/packages/release/bioc/vignettes/ChIPseeker/inst/doc/ChIPseeker.html).

**RNA-Seq analysis**

Total RNA was extracted using an ESciences RNA-Quick Purification Kit (Yi Shan Biotech, Shanghai, China). Library construction and RNA-seq were done by the OE Biotech company (Shanghai, China) with Illumina Nova Seq 6000 (Illumina, San Diego, CA, USA) and the data analyzed. Differentially expressed genes (DEGs) were analyzed by Gene Ontology (GO) annotation enrichment and Kyoto Encyclopedia of Genes and Genomes (KEGG) pathway enrichment with R based on the hypergeometric distribution. The criteria for differential genes were set up with P-value < 0.05 and fold change > 2 or < 0.5.

**CUT&Tag library and sequencing**

Huh7 cells were obtained from the American Type Culture Collection (ATCC; Manassas, VA, USA) and grown according to the supplier's protocol. We used the following antibodies: ANLN Primary antibody. A cleavage under targets and tagmentation (CUT&Tag) library was constructed using Hyperactive TM In-Situ ChIP

Library Prep Kit (TD901, Vazyme, Nanjing, China). In short, after harvesting, counting, and centrifugation (600 g; 3 min; RT), 60-500 000 cells aliquots were washed twice in 500 μL wash buffer by gentle pipetting. Ten microliters of prepared Concanavalin A-coated magnetic beads were added to each sample and incubated at RT for 10 min. Following the protocol, the cells were subjected to successive incubations with the beads, primary antibody, secondary antibody, and Hyperactive PG-TN5/PA-TN5 Transposon, and then fragmented. The fragmented DNA was isolated and amplified by PCR to obtain the library, which was sequenced using PE150 on the Illumina sequencer (Shanghai OE Biotech, China).

**Data processing for CUT&Tag**

We used Fastp v0.20.0 was used to remove adapter and low-quality reads. We then aligned paired-end reads using Bowtie2 version 2.3.4.3 (https://sourceforge.net/projects/bowtie-bio/files/bowtie2/2.3.4.3/) with options: -end-to-end-sensitive. Duplicated reads were removed using Picard v2.18.17 (https://github.com/nigyta/rice_reseq/blob/master/tools/picard-FastqToSam.cwl) with this parameter: REMOVE_DUPLICATES = true. For peak calling, SEACR v1.3 (https://github.com/FredHutch/SEACR) was used with threshold: 0.01. Scatterplots, correlation plots, and heatmaps were displayed using deep Tools v2.27.1 (https://deeptools.readthedocs.io/en/develop/). Annotation of peaks was performed using an R package ChIP seeker v1.12.1 and MEME ChIP v5.0.5 was used to search for the binding site.

## Supplementary Figures

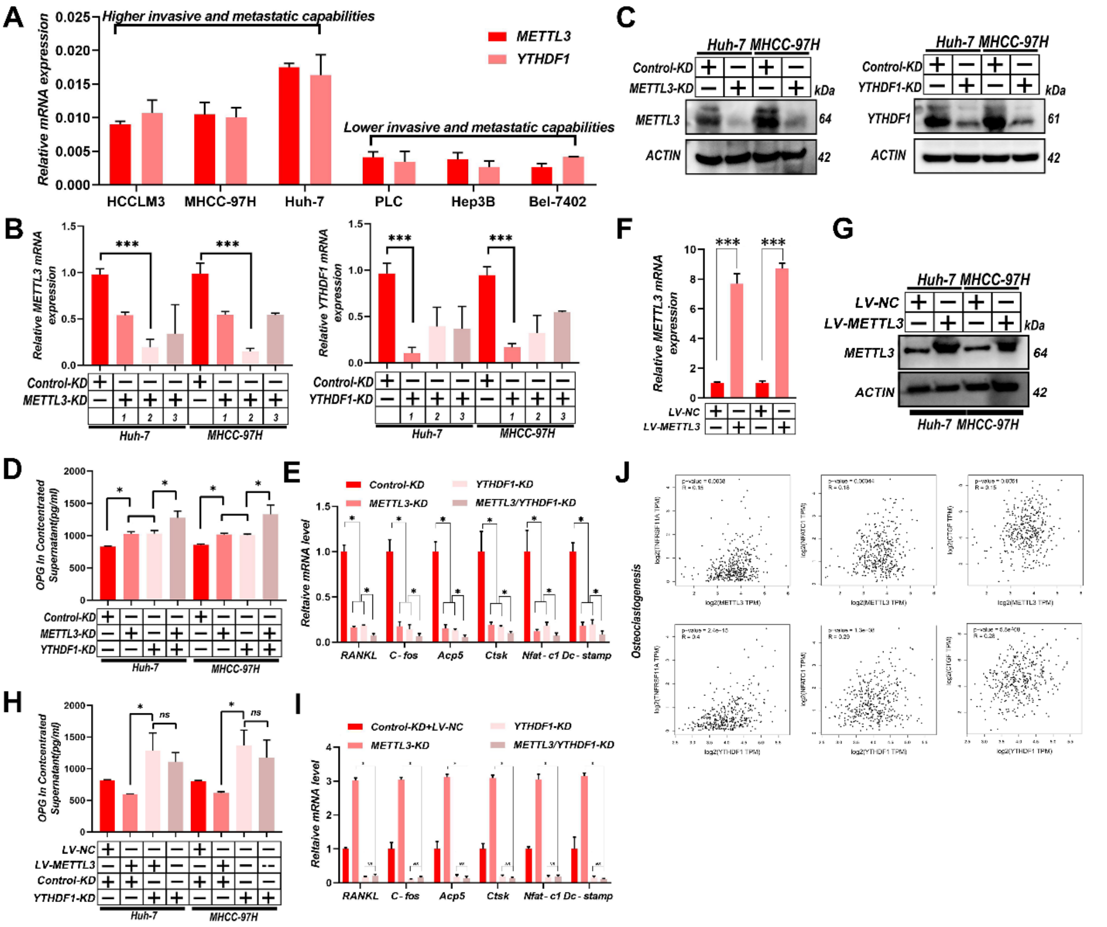


## Supplementary Figure 1.

**A**. *METTL3* and *YTHDF1* mRNA levels in HCC cell lines were assessed using qRTPCR.

**B&C**. qRT-PCR and WB showed the *METTL3 and YTHDF1* mRNA (**B**) and protein (**C**) levels after silencing of *METTL3* and *YTHDF1*, respectively. *METTL3*-KD#2 and *YTHDF1*#1, which induced the most significant knock-down (KD) effect, respectively, was adopted for further study.

**D**. Concentration of *OPG* in the culture medium measured by ELISA from *METTL3/YTHDF1* knockdown and control HCC cell clones.

**E**. Osteoclastogenesis differentiation-related factors mRNA level of RAW264.7 cells with conditioned medium from (**D**) was measured by qRT-PCR treated.

**F&G.** qRT-PCR and WB measurement of *METTL3* mRNA and protein levels in HCC cells with LV-METTL3 or LV-NC.

**H**. Concentration of *OPG* in culture supernatants determined by ELISA from the *METTL3* overexpression and control HCC cells were infected with *YTHDF1*-KD or Control-KD HCC cell clones.

**I**. Osteoclastogenesis differentiation-related factors mRNA level of RAW264.7 cells with conditioned medium from (**H**) was determined by qRT-PCR.

**J.** Correlations of *METTL3 and YTHDF1* with several of osteoclastogenesis-related factors in HCC tissues based on GEPIA database. For (B&F) Paired t-test, (D&E, H&I) one-way ANOVA, (J) Pearson correlation analysis. Error bars represent means ± SD from three independent experiments, $*p < 0.05$; $**p < 0.01$; $***p < 0.001$; NS $p > 0.05$.

qRT-PCR, quantitative reverse transcription-polymerase chain reaction; WB, western blot; HCC, hepatocellular carcinoma; mRNA, messenger RNA; ELISA, enzyme-linked immunosorbent assay; ANOVA, analysis of variance; SD, standard deviation; NS, not significant

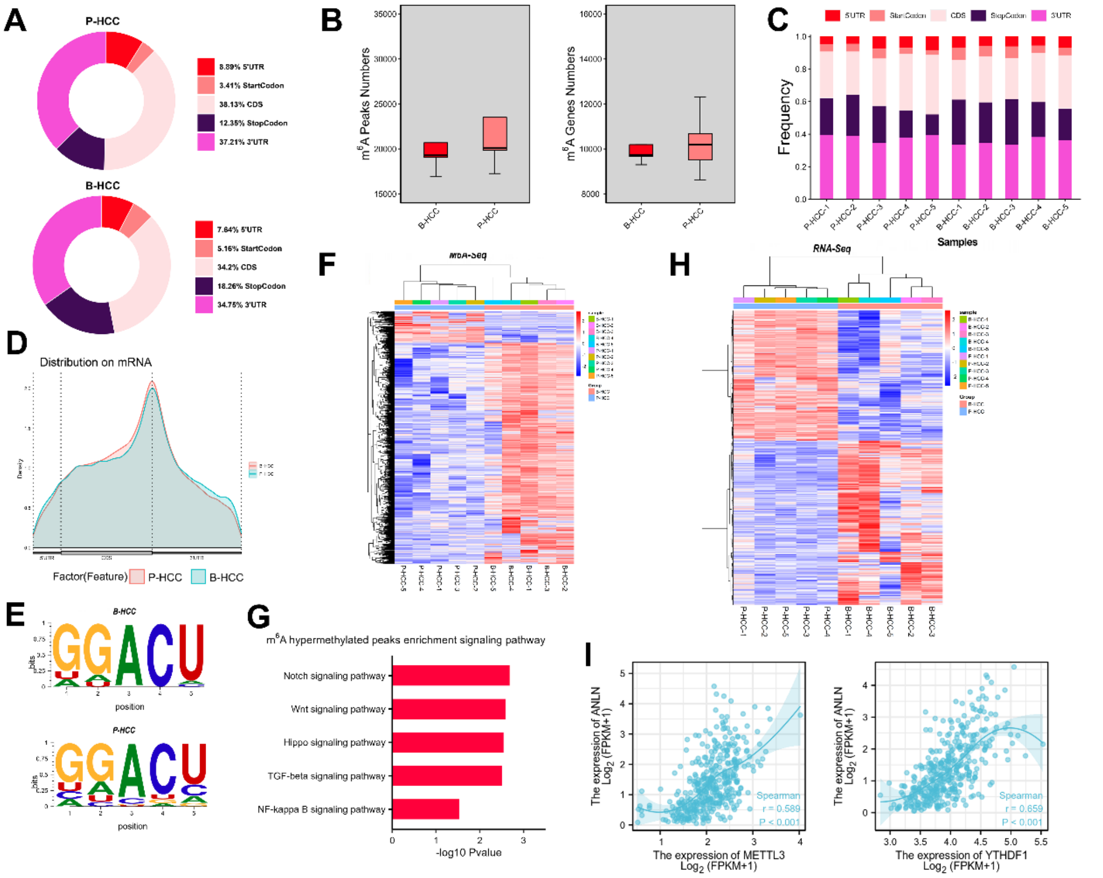


**Supplementary Figure 2.**

**A- E**. RNA categories (**A**), the methylation level change of differential m$^6$A sites in genes (**B**), mRNA region distributions (**C**), the distribution patterns ofm$^6$A methylation peaks in gene structures of mRNA (**D**), and logo plot (**E**) of m$^6$A sites in 5 B-HCC and 5 P-HCC samples.

**F**. Heatmap of changes in methylation levels of differential m$^6$A sites between 5 B-HCC and 5 P-HCC samples.

**G.** Canonical pathway analysis of hypermethylated mRNAs in B-HCC was shown (WNT, Hippo, Notch, TGF-β, and NF-κB).

**H**, Heatmap of differentially expressed mRNAs between 5 B-HCC and 5 P-HCC samples.

**I,** the correlation between *METTL3* and *YTHDF1* with *ANLN* in HCC tissues based on GEPIA database, respectively. For (I) Pearson correlation analysis.

$M^6A$, N6-methyladenosine; mRNA, messenger RNA; B-HCC, hepatocellular carcinoma bone metastasis focus; P-HCC, HCC primary focus; TGF-β, transforming growth factor-β; NF-κB, nuclear factor κB GEPIA, gene expression profile interactive analysis

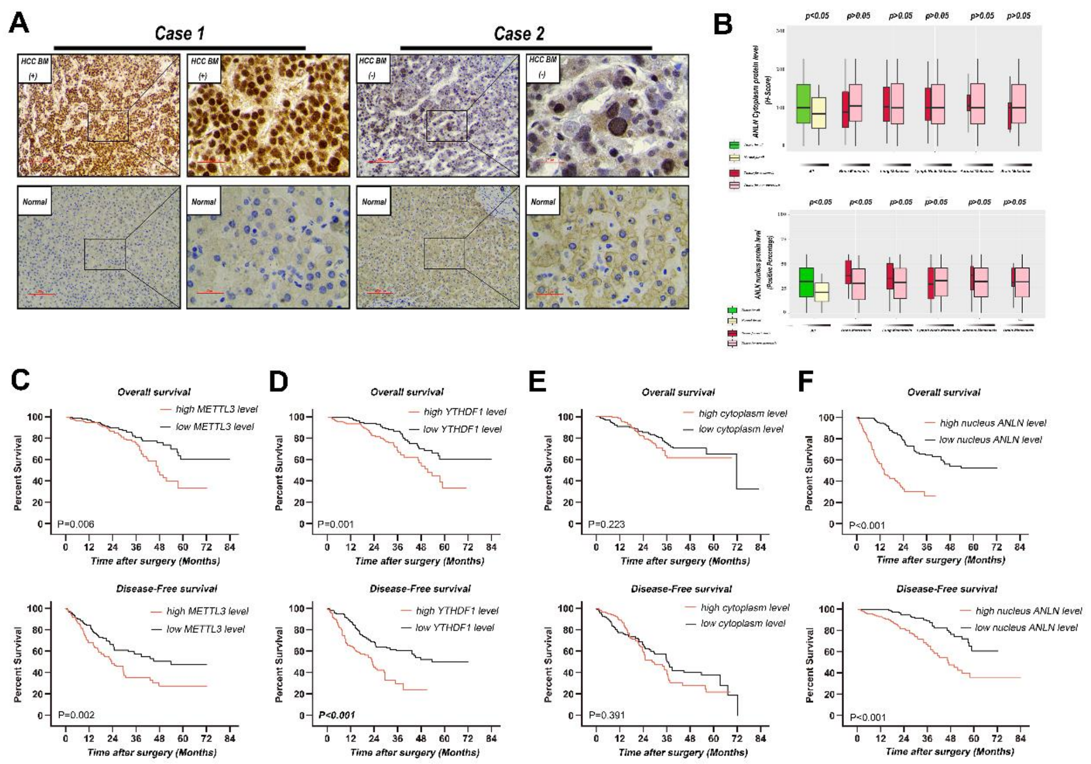


**Supplementary Figure 3.**

**A**. Images of representative *ANLN* staining are shown. Scale bar = 100 µm; Scale bar = 25µm.

**B**. IHC H-Scores of cytoplasm staining and relative nucleus expression of *ANLN* protein from 265 cases.

Of these 265 patients, there were 45 with BM, 53 with lung metastasis, 40 with LNM, 10 with adrenal gland metastasis, and 9 with brain metastases. Note： multiple organ metastases are represented in each relevant organ group.

**C - F**. Kaplan-Meier OS and DFS curves for 265 HCC patients expressing high or low levels of *METTL3* (**C**), *YTHDF1* (**D**), nucleus *ANLN* (**E**) and cytoplasm *ANLN* (**F**) stain. (B) Unpaired t-test, (C-F) log-rank test. Error bars represent means ± SD from three independent experiments, *p < 0.05; **p < 0.01; ***p < 0.001; NS p > 0.05.

HCC, hepatocellular carcinoma; mRNA, messenger RNA; OS, overall survival; DFS, disease-free survival; SEM, standard error of the mean; NS, not significant

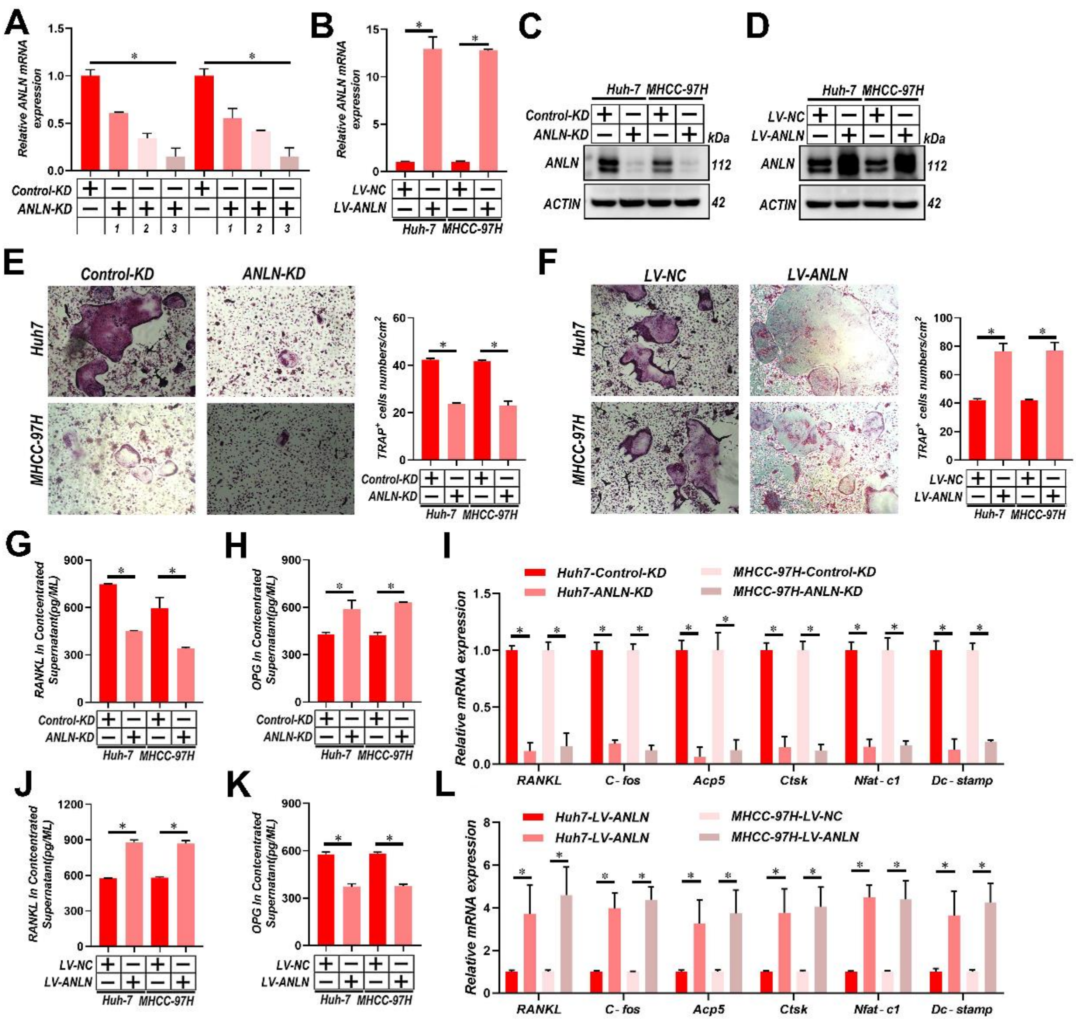

**Supplementary Figure 4.**

**A&B.** *ANLN* mRNA levels measured by qRT-PCR from indicated HCC cells. *ANLN*-KD#3 which induced the most significant knock-down (KD) effect, respectively, was adopted for further study.

**C&D**. Confirmation of *ANLN* knockdown (KD; *ANLN*-KD), and over-expression (LV-*ANLN*) in HCC cell lines were performed by WB.

**E&F**. Osteoclast differentiation assays were co-cultured with conditioned media from the HCC cells group in (**C&D**) containing M-CSF (25 ng/mL) and *RANKL* (100 ng/mL), magnification × 200.

**G&J**. Concentrations of *RANKL* in culture supernatants determined by ELISA from each group HCC cell.

**H&K**. Concentrations of *OPG* in culture supernatants determined by ELISA from each group HCC cell.

**I&L**. Osteoclastogenesis differentiation-related factors mRNA level of RAW264.7 cells with conditioned medium from (**F&G**) was measured by qRT-PCR treated.

For (A & B, G-L) Paired t-test. Error bars represent means ± SD from three independent experiments, *$p < 0.05$; **$p < 0.01$; ***$p < 0.001$; NS $p > 0.05$.

qRT-PCR, quantitative reverse transcription-polymerase chain reaction; WB, western blot; BLI, BLI, bioluminescence imaging; M-CSF, macrophage colony-stimulating factor; HCC, hepatocellular carcinoma; mRNA, messenger RNA; ELISA, enzyme-linked immunosorbent assay; SD, standard deviation

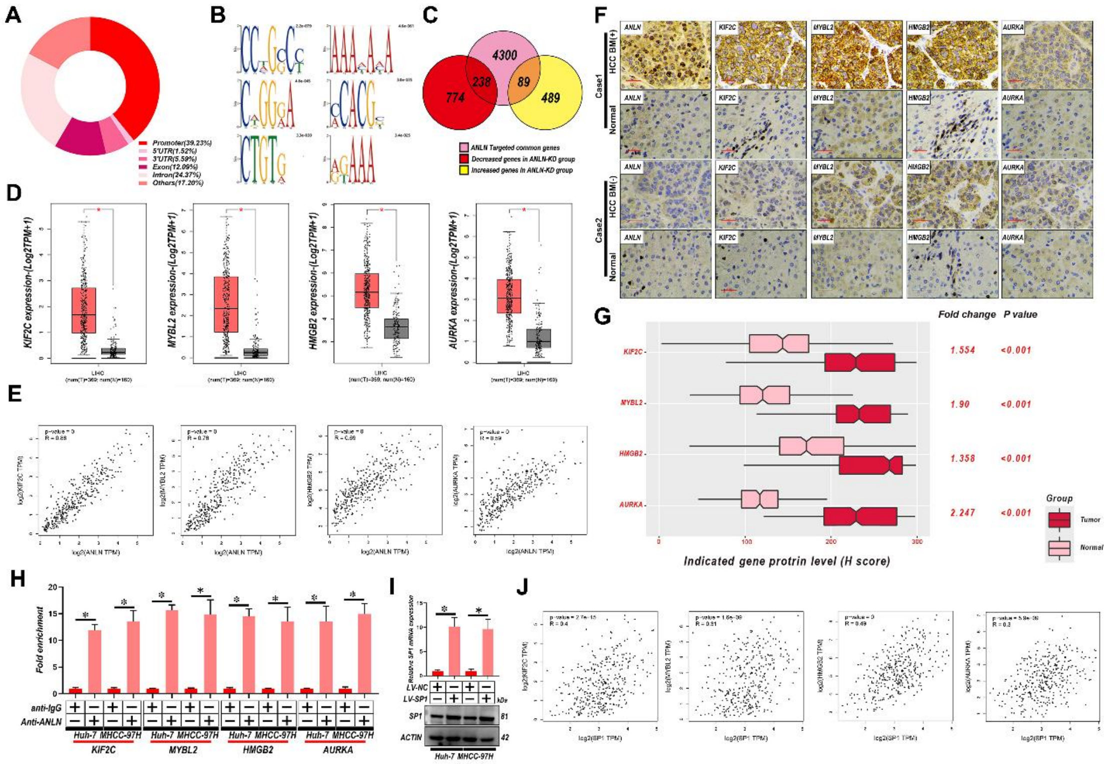

**Supplementary Figure 5.**

**A**. CUT&Tag seq dataset for *ANLN* in Huh7 cells produced using anti-ANLN antibody. The pie chart indicates the genome-wide distribution of ANLN occupancy in promoter, exon, intron, and intergenic regions in Huh7 cells after peak calling.

**B**. *ANLN*-binding consensus in Huh7 cells, Top 6.

**C**. Venn diagram of *ANLN*-regulated genes (Control vs. *ANLN*-KD: Blue circle, down-regulated; Green circle, up-regulated) and *ANLN*-targeted genes (Blue circle).

**D**. Expression of *KIF2C*, *MYBL2*, *HMGB2*, and *AURKA* transcript expression in HCC and normal tissues were performed in the GEPIA database.

**E**. The correlation between *ANLN* and *KIF2C*、*MYBL2*、*HMGB2*、and *AURKA* in HCC based on GEPIA database, respectively.

**F**. Representative IHC stains of *KIF2C, MYBL2, HMGB2,* and *AURKA* in matched 265 HCC and adjacent normal tissues. Scale bars, 200 µm, scale bars, 25 µm.

**G**. Histochemistry score (H-Score) was used to calculate the relative protein expression of *KIF2C*, *MYBL2*, *HMGB2*, and *AURKA* in 265 paired HCC and adjacent normal tissues.

**H**. Hun7 and MHCC-97H were harvested and analyzed by ChIP assays with an anti-ANLN antibody and qPCR. The figure shows ChIP-qPCR assessments of *ANLN* enrichment at the *KIF2C*, *MYBL2*, *HMGB2*, and *AURKA* promoter regions.

**I.** qRT-PCR and WB measurements of *SP1* mRNA and protein levels in HCC cells with LV-NC or LV-*SP1*.

**J**. The correlation between *SP1* and *KIF2C*、*MYBL2*、*HMGB2*、and *AURKA* in HCC based on GEPIA database, respectively,

For (D, G, and H & I) Paired t-test, (E, J) Pearson correlation analysis. Error bars represent means ± SD from three independent experiments, *p < 0.05; **p < 0.01; ***p < 0.001; NS p > 0.05.

CUT&Tag, cleavage under targets and tagmentation; HCC, hepatocellular carcinoma; GEPIA, gene expression profile interactive analysis; IHC, immunohistochemical; qRT-PCR, quantitative reverse transcription-polymerase chain reaction; WB, western blot; SD, standard deviation; NS, not significant

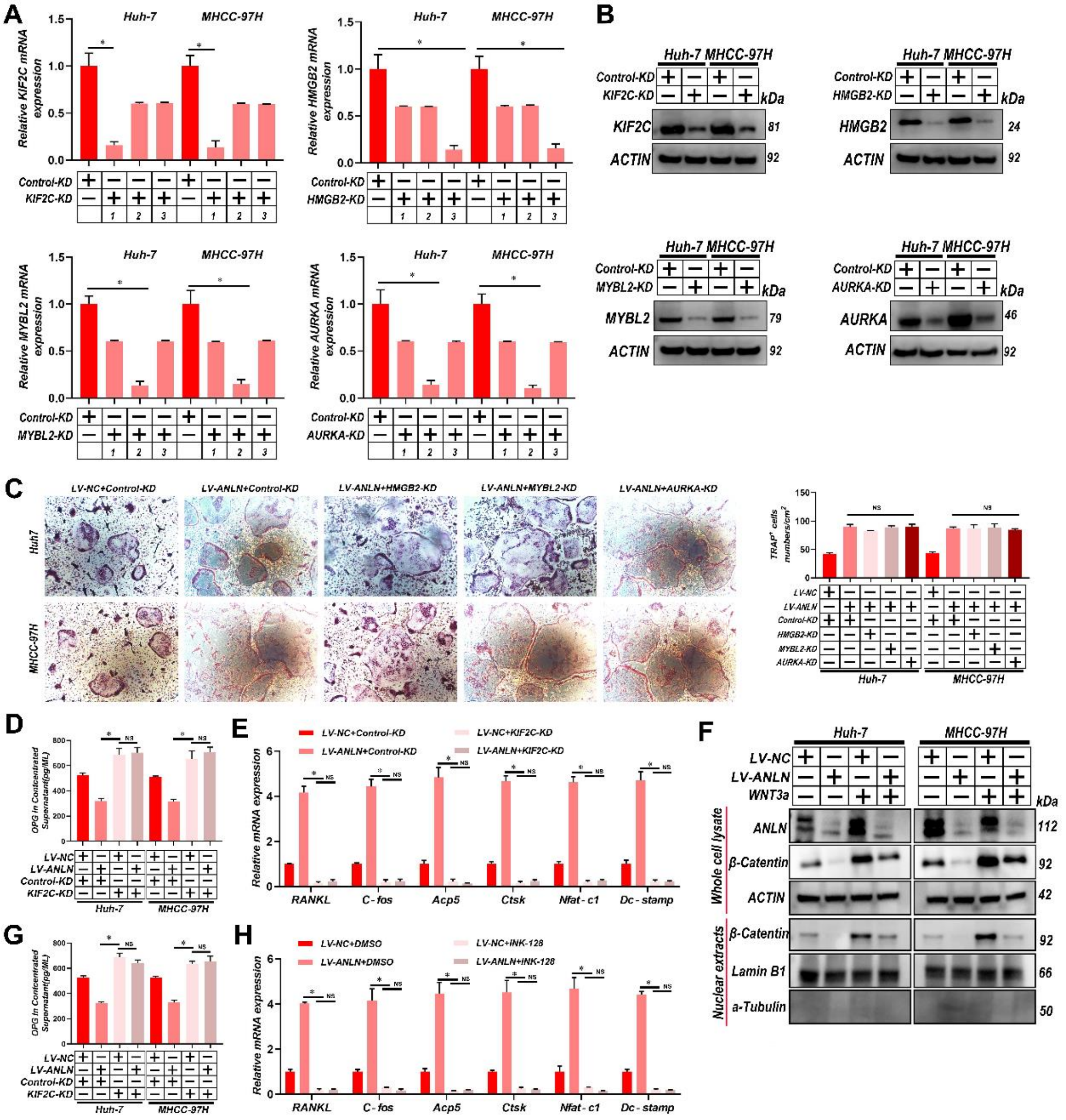


**Supplementary Figure 6.**

**A&B**. Confirmation of *KIF2C*, *HMGB2*, *MYBL2*, and *AURKA* knockdown (KD) in HCC cell lines were performed by qRT-PCR and WB. *KIF2C*-KD#1, HMGB2-KD#3, *MYBL2#2* and *AURKA#2*, which induced the most significant knock-down (KD) effect, respectively, was adopted for further study.

**C**. Osteoclast differentiation assays were co-cultured with conditioned media from the indicated HCC cells group containing M-CSF (50 ng/mL) and *RANKL* (100 ng/mL), magnification × 200.

**D**. Concentration of *OPG* in the culture medium measured by ELISA from each group HCC cell.

**E.** Osteoclastogenesis differentiation-related factor mRNA levels in RAW264.7 cells with conditioned medium from (**D**), determined by qRT-PCR.

**F.** WB showing levels of marker proteins (Lamin B1: nuclear marker, α-tubulin: cytoplasmic marker).

**G**. *ANLN* overexpression and control HCC cells were treated with or without INK-128, concentration of *OPG* in the culture medium measured by ELISA from each group HCC cell.

**H.** Osteoclastogenesis differentiation-related factors mRNA level of RAW264.7 cells with conditioned medium from (**G**), determined by qRT-PCR.

For (A) Paired t-test, (C-E, G&H) one-way ANOVA. Error bars represent means ± SD from three independent experiments, *$p < 0.05$; **$p < 0.01$; ***$p < 0.001$; NS $p > 0.05$.

HCC, hepatocellular carcinoma; qRT-PCR, quantitative reverse transcription-polymerase chain reaction; WB, western blot; M-CSF, macrophage colony-stimulating factor; ANOVA, analysis of variance; SD, standard deviation; NS, not significant

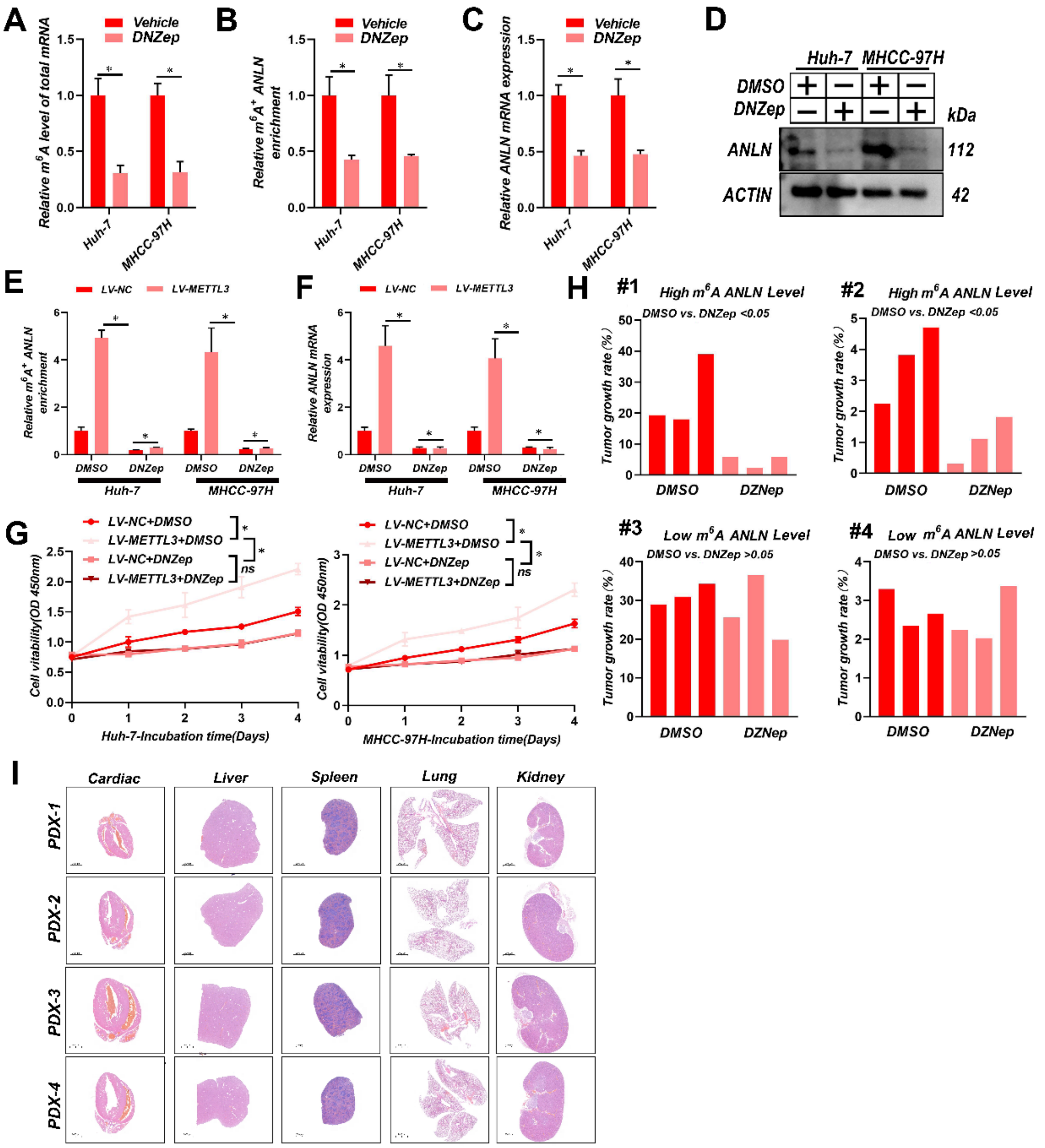


**Supplementary Figure 7.**

**A**. m$^6$A levels in HCC cells treated with vehicle or DZNep (10 mM) assessed using LC-MS/MS.

**B.** MeRIP-qPCR analysis was employed to assessment the *ANLN* m$^6$A modifications in HCC cells treated with vehicle or DZNep (10 mM).

**C&D**. *ANLN* mRNA and protein levels in HCC cells treated with vehicle or DZNep (10 mM), shown by qRT-PCR and WB, respectively.

**E.** MeRIP-qPCR assessment of *ANLN* m$^6$A modifications in HCC cells with *LV-NV or LV-METTL3*, and treated with vehicle or DZNep (10 mM).

**F.** *ANLN* mRNA levels determined by qRT-PCR in HCC cells with LV-NV or *LV-METTL3* and treated with vehicle or DZNep (10 mM).

**G.** The *in vitro* CCK8 assays in HCC cells with *LV-NV or LV-METTL3*, and treated with vehicle or DZNep (10 mM).

**H.** Growth of xenograft tumors. Vehicle (DMSO)-treated PDX were used as controls.

**I.** H&E staining of mice organs was performed from(**H**).

For (A-C, G, H) Paired t-test, (E and F) one-way ANOVA. Error bars represent means ± SD from three independent experiments, *p < 0.05; **p < 0.01; ***p < 0.001; NS p > 0.05.

$M^6A$, N6-methyladenosine; HCC, hepatocellular carcinoma; qRT-PCR, quantitative reverse transcription-polymerase chain reaction; WB, western blot; ANOVA, analysis of variance; SD, standard deviation; NS, not significant; MeRIP-qPCR, methylated RNA immunoprecipitation-quantitative reverse transcription-polymerase chain reaction; DZNep, 3-deazaneplanocin A; LC-MS/MS, liquid chromatography tandem mass spectrometry

**Supplementary Tables**

**Table S1. Summary of clinico-pathologic characteristics of 29 HCC patients.**

| Group | Patient ID | Gender | Age | serum AFP, ng/mL | Tumor diameter, cm | Tumor number | Vascular invasion | TBIL, µmol/L | ALT, U/L | PT, s | PLT, 10^9/L |
|---|---|---|---|---|---|---|---|---|---|---|---|
| advantage-stage HCC | 1 | Male | 42 | 1210 | 2.7 | 1 | Presence | 9.1 | 10.6 | 12.4 | 146 |
| | 2 | Male | 55 | 1210 | 4.8 | 1 | Presence | 16.6 | 48.8 | 13 | 109 |
| | 3 | Male | 46 | 70 | 4.8 | 2 | Presence | 10.9 | 113.3 | 12.8 | 154 |
| | 4 | Male | 41 | 1210 | 4.3 | 1 | Presence | 10.8 | 31.2 | 13.2 | 212 |
| | 5 | Male | 60 | 1210 | 5 | 2 | Presence | 18.6 | 44.5 | 12.2 | 195 |
| | 6 | Male | 42 | 2.3 | 4.2 | 1 | Presence | 15.6 | 33.3 | 11.6 | 100 |
| | 7 | Male | 50 | 2.1 | 4.9 | 1 | Presence | 13.4 | 20.2 | 12 | 175 |
| | 8 | Male | 37 | 14.1 | 2.8 | 1 | Presence | 19.6 | 18.5 | 10.7 | 82 |
| | 9 | Male | 60 | 1210 | 4 | 1 | Presence | 12.3 | 80.9 | 12.5 | 122 |
| | 10 | Male | 40 | 4.9 | 3.2 | 1 | Absence | 24 | 42.8 | 14.4 | 31 |
| HCC with metastasis (non-bone) | 1 | Male | 44 | 72.5 | 2.4 | 1 | Absence | 21.4 | 52 | 12.1 | 176 |
| | 2 | Male | 27 | 3.4 | 3.2 | 1 | Absence | 9.9 | 34.1 | 12 | 214 |
| | 3 | Male | 51 | 5.6 | 3.3 | 1 | Absence | 15.1 | 23.9 | 11 | 124 |
| | 4 | Male | 39 | 13.2 | 3.2 | 1 | Absence | 11.7 | 39.9 | 11.8 | 323 |
| | 5 | Male | 51 | 3.5 | 3.3 | 1 | Absence | 21.1 | 69.3 | 10.4 | 156 |
| | 6 | Male | 40 | 474 | 1.8 | 1 | Absence | 13.4 | 40.3 | 12.5 | 101 |
| | 7 | Male | 41 | 325 | 1.3 | 1 | Absence | 10.5 | 35.4 | 11.9 | 143 |
| | 8 | Male | 54 | 12.2 | 5 | 1 | Absence | 6 | 26.8 | 10.7 | 229 |
| | 9 | Male | 29 | 15.5 | 2.5 | 2 | Absence | 19 | 44 | 12.3 | 165 |
| | 10 | Male | 42 | >1210 | 8 | 1 | Presence | 22.3 | 58 | 12.5 | 137 |
| | 11 | Male | 53 | 629.7 | 5 | >3 | Absence | 2.4 | 22 | 10.9 | 186 |
| | 12 | Male | 48 | 9 | 11.6 | 1 | Absence | 13.1 | 44 | 12.7 | 216 |
| | 13 | Female | 53 | 49.4 | 8 | 1 | Absence | 12 | 42 | 10.6 | 141 |
| HCC with BM | 1 | Male | 64 | 143 | 11 | 2 | Absence | 3.8 | 16 | 11.5 | 287 |
| | 2 | Male | 66 | 3.6 | 10 | 1 | Presence | 11.5 | 45 | 10.6 | 173 |
| | 3 | Male | 29 | 53778 | 8.6 | 1 | Absence | 18.8 | 45 | 11.3 | 157 |
| | 4 | Male | 43 | 426 | 15.3 | 2 | Absence | 23.7 | 27 | 14.1 | 271 |
| | 5 | Male | 31 | 9992 | 8.9 | 1 | Absence | 13.7 | 43 | 11.4 | 126 |
| | 6 | Male | 67 | 4.6 | 10 | >3 | Absence | 12.3 | 65 | 9.8 | 276 |

HCC, hepatocellular carcinoma; BM HCC, HCC with bone mestasis; TBIL, total bilirubin; ALT, alanine aminotransferase; PT, prothrombin time; PLT, platelet; HBeAg, hepatitis Be antigen

**Table S2. Summary of clinico-pathologic characteristics IHC Cohort (n=265).**

| Clinico-pathologic characteristic | | N |
|---|---|---|
| **Gender** | Female | 35 (13.2%) |
| | Male | 230 (86.8%) |
| **Age, year** | **≥55** | 88 (33.2%) |
| | **<55** | 177 (66.8%) |
| **HBsAg** | Negative | 4 (1.51%) |
| | Positive | 261 (98.5%) |
| **HBV DNA, cpa/mL** | ≥ 1000 | 165 (62.3%) |
| | < 1000 | 100 (37.7%) |
| **AFP, ng/mL** | ≥20 | 72 (27.2%) |
| | <20 | 193 (72.8%) |
| **Vascular invasion** | No | 176 (66.4%) |
| | yes | 89 (33.6%) |
| **Tumor diameter, cm** | ≥5 | 47 (17.7%) |
| | <5 | 218 (82.3%) |
| **Tumor number** | Single | 202 (76.2%) |
| | Multiple | 63(21.5%) |
| **Tumor Capsule** | Absence | 59 (22.3%) |
| | Presence | 206 (77.7%) |
| **Liver Cirrhosis** | No | 92 (34.7%) |
| | Yes | 173 (65.3%) |

TBIL, total bilirubin; ALT, alanine aminotransferase; PT, prothrombin time; PLT, platelet; HBeAg, hepatitis Be antigen; IHC Cohort, immunohistochemistry cohort

**Table S3. $m^6A$ and transcript level of $m^6A$ regulate genes in 6 HCC with BM (BM HCC), 13 HCC with metastasis (non-bone), and 10 advantage-stage HCC, as assessed using qRT-PCR analysis**

| Group | Metastasis(bone+) vs. Metastasis(bone-) | | Metastasis(bone-) vs. no-Metastasis | | Metastasis(bone+) vs. non-Metastasis | |
|---|---|---|---|---|---|---|
| | **Foldchange** | **p-value** | **Foldchange** | **p-value** | **Foldchange** | **p-value** |
| $m^6A$ | 1.612266238 | 0.019906515 | 1.718952882 | 0.003597039 | 1.066171853 | 0.669931 |
| METTL3 | 2.76697575 | 0.000150521 | 2.46075325 | 0.000663556 | 0.889329533 | 0.418831 |
| METTL14 | 0.916997687 | 0.657353693 | 1.160382144 | 0.463178941 | 1.265414472 | 0.039536 |
| WTAP | 0.906814899 | 0.604690641 | 0.901006863 | 0.582367949 | 0.993595125 | 0.96176 |
| KIAA1429 | 0.434563829 | 0.011346058 | 0.758315438 | 0.154132577 | 1.745003585 | 0.011057 |
| ALKBH5 | 0.179233225 | 1.89781E-06 | 0.727066888 | 0.011890817 | 4.056540803 | 3.07E-08 |
| FTO | 0.546718462 | 0.055294424 | 1.296767645 | 0.226639943 | 2.371894761 | 0.002514 |
| YTHDF1 | 2.588847529 | 2.34341E-09 | 2.717462261 | 2.65911E-07 | 1.049680304 | 0.66467 |
| YTHDF2 | 3.54162141 | 0.044841223 | 1.621534816 | 0.326483722 | 0.457854149 | 0.000274 |
| YTHDF3 | 0.580681912 | 0.15089313 | 0.868910808 | 0.344686312 | 1.496362794 | 0.137908 |

HCC, hepatocellular carcinoma; BM HCC, HCC with bone mestasis

**Table S4. List of genes with a significant m6A hypermethylation and up-regulated expression levels in 5 B-HCC vs. 5 P-HCC samples.**

| Gene | $m^6$A Peak | | mRNA | |
|---|---|---|---|---|
| | Diff.Log2.FC | p-value | log2FoldChange | p-value |
| GPC3 | 12.4572754 | 0.000211266 | 5.423352434 | 3.15133E-08 |
| MAGEA6 | 11.45155631 | 0.001342733 | 10.53480235 | 0.000499434 |
| TRIM71 | 10.9129911 | 5.68529E-06 | 9.077473284 | 2.94033E-09 |
| MAGEA12 | 10.87022834 | 0.002055018 | 10.25435875 | 0.000703119 |
| REG3A | 10.70888244 | 0.0001944 | 9.420241988 | 9.38696E-08 |
| MAFA | 10.24393369 | 0.000829402 | 8.032313619 | 0.000451791 |
| KREMEN2 | 9.5471569 | 0.000242909 | 7.923597321 | 9.69889E-08 |
| NAA11 | 9.22316457 | 0.003552641 | 23.08525504 | 2.40693E-14 |
| BPIFB2 | 8.883203746 | 0.000528499 | 8.471042175 | 0.000321221 |
| MAGEA10 | 8.857325058 | 0.001377232 | 22.91360668 | 3.70325E-14 |
| MEP1A | 8.775497385 | 0.001471962 | 6.305677358 | 0.000259655 |
| TICRR | 8.672902978 | 0.000224039 | 4.257441837 | 3.87697E-06 |
| COL11A1 | 8.586398697 | 0.000700843 | 6.294084535 | 4.29543E-08 |
| C20orf204 | 8.531011937 | 0.000237157 | 5.821451323 | 4.38937E-12 |
| CCR8 | 8.411682056 | 0.000213738 | 4.623170612 | 0.000246411 |
| KCNF1 | 8.142172121 | 0.001363504 | 5.698712402 | 0.000248883 |
| PRAME | 7.925111921 | 0.000524497 | 7.152787107 | 7.40783E-05 |
| NOTUM | 7.895103408 | 0.003480605 | 4.552731018 | 0.000120146 |
| ZIC2 | 7.886597017 | 0.002507065 | 8.359057073 | 9.83892E-09 |
| GPC3 | 7.874970119 | 7.45213E-05 | 5.423352434 | 3.15133E-08 |
| ZIC5 | 7.857448977 | 0.004005667 | 6.379082207 | 0.000801129 |
| SLC22A31 | 7.561087301 | 0.000435186 | 5.133470547 | 4.48504E-05 |
| COL11A1 | 7.55149037 | 0.002518633 | 6.294084535 | 4.29543E-08 |
| NPFFR2 | 7.533877749 | 0.002838726 | 7.710536855 | 2.78447E-05 |
| B4GALNT4 | 7.465075936 | 0.001080227 | 4.492440609 | 4.00515E-05 |
| ZIC2 | 7.37341852 | 8.82512E-05 | 8.359057073 | 9.83892E-09 |
| IGFBPL1 | 7.301867939 | 0.000369771 | 4.061134838 | 4.7178E-06 |
| GABRQ | 7.194718334 | 0.012052278 | 6.045089432 | 2.16471E-05 |
| PITX1 | 7.081516843 | 0.000639509 | 8.557427502 | 3.17882E-09 |
| SIX4 | 6.993405653 | 0.000268619 | 4.846512979 | 4.59561E-05 |
| SPINK5 | 6.978431652 | 0.01400182 | 5.532768438 | 1.33822E-06 |
| CA12 | 6.917707128 | 0.000864271 | 5.457372325 | 4.10032E-05 |
| CRYBG2 | 6.83088253 | 0.002418559 | 5.109525054 | 9.24401E-09 |
| MEP1A | 6.808561422 | 0.016594478 | 6.305677358 | 0.000259655 |
| C5orf46 | 6.808506248 | 0.009824018 | 6.118909628 | 1.82684E-05 |
| MEP1A | 6.78997516 | 0.00370127 | 6.305677358 | 0.000259655 |
| PRAME | 6.664013245 | 0.003650552 | 7.152787107 | 7.40783E-05 |
| NKD1 | 6.513261563 | 0.004344455 | 4.919387484 | 1.75791E-05 |
| CENPF | 6.395071763 | 0.001157275 | 4.092018883 | 8.71647E-11 |
| CCNO | 6.215516109 | 0.004405498 | 4.496338689 | 0.00040676 |

| | | | | |
|---|---|---|---|---|
| CACNG4 | 6.193455658 | 0.000498446 | 5.42749299 | 7.04038E-05 |
| CDC25C | 6.179229261 | 0.002862171 | 4.576076152 | 5.93672E-10 |
| NAA11 | 6.175244457 | 0.00644871 | 23.08525504 | 2.40693E-14 |
| EXO1 | 6.137457843 | 0.000902958 | 4.130487664 | 2.96599E-10 |
| NKD1 | 6.122121231 | 0.005873604 | 4.919387484 | 1.75791E-05 |
| NKD1 | 6.02914564 | 0.004518617 | 4.919387484 | 1.75791E-05 |
| E2F8 | 5.936927922 | 0.000510963 | 4.048552813 | 2.82265E-11 |
| TOP2A | 5.930908096 | 0.015241869 | 4.060501119 | 6.67813E-12 |
| PPP2R2C | 5.815300062 | 0.001435768 | 7.423797575 | 1.22335E-06 |
| SLC22A11 | 5.7859506 | 0.003213869 | 4.031917208 | 8.58607E-05 |
| HOXA13 | 5.783308637 | 0.002224118 | 5.063294181 | 0.00044068 |
| HOXA10 | 5.743600955 | 0.005796136 | 6.969466403 | 3.3998E-07 |
| PPP2R2C | 5.681542246 | 0.002257948 | 7.423797575 | 1.22335E-06 |
| HOXA13 | 5.623458111 | 0.007833321 | 5.063294181 | 0.00044068 |
| NOTUM | 5.621608789 | 0.002287596 | 4.552731018 | 0.000120146 |
| KCNJ6 | 5.613499064 | 0.001400604 | 5.909640603 | 4.38419E-06 |
| DEPDC1 | 5.477321024 | 0.000787312 | 4.659499665 | 1.29432E-06 |
| CENPF | 5.476089347 | 3.07184E-05 | 4.092018883 | 8.71647E-11 |
| OTX1 | 5.461838165 | 0.006883836 | 5.008753683 | 0.00013086 |
| TOP2A | 5.448307794 | 0.004007511 | 4.060501119 | 6.67813E-12 |
| DEPDC1 | 5.428245877 | 0.002354101 | 4.659499665 | 1.29432E-06 |
| CCR8 | 5.356524967 | 0.000766031 | 4.623170612 | 0.000246411 |
| CENPF | 5.326259472 | 2.8E-05 | 4.092018883 | 8.71647E-11 |
| GPC3 | 5.315422219 | 0.002418674 | 5.423352434 | 3.15133E-08 |
| CENPF | 5.30020122 | 0.000179541 | 4.092018883 | 8.71647E-11 |
| NRCAM | 5.288605795 | 0.004783961 | 4.346381187 | 2.48314E-05 |
| SLC2A1 | 5.052946623 | 0.000780508 | 4.020640443 | 7.1024E-05 |
| SFN | 5.044340256 | 0.000396558 | 4.909022212 | 5.40845E-08 |
| MUC13 | 4.984176502 | 0.001020376 | 5.559976908 | 1.56519E-14 |
| HOXA13 | 4.908245774 | 0.016237031 | 5.063294181 | 0.00044068 |
| CENPF | 4.896819619 | 0.005721252 | 4.092018883 | 8.71647E-11 |
| UBE2C | 4.793578261 | 0.008115567 | 4.835442051 | 5.42776E-09 |
| NXPH4 | 4.789619414 | 0.013010984 | 4.637854416 | 1.71746E-05 |
| CENPF | 4.753549882 | 0.000201064 | 4.092018883 | 8.71647E-11 |
| EXO1 | 4.712761645 | 0.049561479 | 4.130487664 | 2.96599E-10 |
| AUNIP | 4.66709518 | 0.004316035 | 4.476846181 | 4.91798E-05 |
| CENPF | 4.663038328 | 0.003574309 | 4.092018883 | 8.71647E-11 |
| UHRF1 | 4.648932259 | 0.000434374 | 4.110385646 | 1.72976E-12 |
| PRRX1 | 4.617118659 | 0.007840701 | 4.357241279 | 2.93366E-07 |
| CENPF | 4.611657775 | 0.000131089 | 4.092018883 | 8.71647E-11 |
| TOP2A | 4.558414279 | 0.002101467 | 4.060501119 | 6.67813E-12 |
| GINS1 | 4.51603461 | 0.004565491 | 4.184492563 | 3.70177E-10 |
| TICRR | 4.483040539 | 0.007871365 | 4.257441837 | 3.87697E-06 |
| NKD1 | 4.470241507 | 0.035929067 | 4.919387484 | 1.75791E-05 |

| | | | | |
|---|---|---|---|---|
| CENPF | 4.434391484 | 0.017796527 | 4.092018883 | 8.71647E-11 |
| ZBED2 | 4.357261373 | 0.004217319 | 6.592973596 | 1.04118E-05 |
| MELK | 4.342726821 | 0.004113781 | 4.184462107 | 1.1564E-10 |
| GP2 | 4.314249193 | 0.045398181 | 5.559266596 | 2.33484E-05 |
| EXO1 | 4.313968336 | 0.003942195 | 4.130487664 | 2.96599E-10 |
| NCAPG | 4.182223475 | 0.00611603 | 4.037536103 | 1.77113E-09 |
| KIF15 | 4.130441334 | 0.016572759 | 4.209217227 | 8.43226E-10 |
| EBF2 | 4.056045632 | 0.036089438 | 4.336271729 | 6.55617E-06 |
| NEK2 | 4.031420154 | 0.010663666 | 4.891126562 | 1.27471E-09 |
| TICRR | 3.982143633 | 0.02073617 | 4.257441837 | 3.87697E-06 |
| TOP2A | 3.887706419 | 0.013359524 | 4.060501119 | 6.67813E-12 |
| CENPA | 3.885426596 | 0.003040713 | 5.027462865 | 2.01019E-07 |
| GINS1 | 3.876862692 | 0.031751657 | 4.184492563 | 3.70177E-10 |
| MYBL2 | 3.854692021 | 0.031707992 | 5.379512273 | 4.48175E-10 |
| HS6ST2 | 3.84475521 | 0.028364567 | 4.622128617 | 0.000544563 |
| TICRR | 3.818082815 | 0.017431726 | 4.257441837 | 3.87697E-06 |
| SIX4 | 3.794612887 | 0.01976303 | 4.846512979 | 4.59561E-05 |
| MUC13 | 3.782292079 | 0.017404635 | 5.559976908 | 1.56519E-14 |
| TOP2A | 3.731049918 | 0.00652102 | 4.060501119 | 6.67813E-12 |
| TOP2A | 3.721243086 | 0.025913883 | 4.060501119 | 6.67813E-12 |
| ANLN | 3.714875427 | 0.015496257 | 4.129812832 | 6.56595E-09 |
| SLC9A3 | 3.618003837 | 0.029739498 | 4.440875171 | 0.000501284 |
| RHBDL3 | 3.468209427 | 0.037252031 | 4.19325615 | 0.000288592 |
| TOP2A | 3.368723298 | 0.019055311 | 4.060501119 | 6.67813E-12 |
| CENPF | 3.196553013 | 0.038648494 | 4.092018883 | 8.71647E-11 |

$M^6A$, N6-methyladenosine; HCC, hepatocellular carcinoma

**Table S5. $m^6A$ and transcript level of 68 genes in 6 HCC with BM (BM HCC), 13 HCC with metastasis (non-bone), and 10 advantage-stage HCC, as assessed using MeRIP-PCR analysis**

| Group | Metastasis(bone+) vs. Metastasis(bone-) | | Metastasis(bone-) vs. no-Metastasis | | Metastasis(bone+) vs. non-Metastasis | |
|---|---|---|---|---|---|---|
| **MeRIP-PCR** | **Foldchange** | **p-value** | **Foldchange** | **p-value** | **Foldchange** | **p-value** |
| ANLN | 4.41097119 | 6.9589E-08 | 1.00578077 | 0.98084249 | 4.43647 | 3.0587E-08 |
| GP2 | 1.11520815 | 0.20155093 | 2.61502154 | 6.8589E-05 | 2.91629333 | 3.5986E-07 |
| NKD1 | 1.23510136 | 0.36843225 | 2.35671077 | 0.00493217 | 2.91077667 | 2.7247E-06 |
| OTX1 | 1.39898955 | 0.22272577 | 1.99309923 | 0.00635125 | 2.788325 | 4.355E-05 |
| EXO1 | 1.23188751 | 0.46290236 | 2.06489769 | 0.01183743 | 2.54372167 | 5.5243E-05 |
| PRAME | 1.19344668 | 0.08479948 | 1.08056077 | 0.28264937 | 1.28959167 | 0.0001035 |
| CA12 | 1.21506517 | 0.03034816 | 1.05525615 | 0.53594539 | 1.282205 | 0.00013485 |
| KIF15 | 1.10603128 | 0.87659103 | 2.64722923 | 5.3948E-05 | 2.92791833 | 0.00015582 |
| MUC13 | 0.99901214 | 0.80093964 | 2.16941308 | 0.00391144 | 2.16727 | 0.00034175 |
| NEK2 | 1.12636041 | 0.5217231 | 2.70688077 | 0.00049488 | 3.04892333 | 0.00037442 |
| MELK | 1.15475851 | 0.7904497 | 2.16800308 | 0.00530343 | 2.50352 | 0.00041439 |
| GABRQ | 1.09308751 | 0.17976759 | 1.20845615 | 0.00634012 | 1.32094833 | 0.00044973 |
| SFN | 1.32418518 | 0.29430388 | 1.70929769 | 0.02715035 | 2.26342667 | 0.00058316 |
| UHRF1 | 1.2511435 | 0.33011492 | 2.20213615 | 0.00303816 | 2.75518833 | 0.0005843 |
| SLC22A11 | 0.86229375 | 0.63256601 | 2.85834923 | 0.00018508 | 2.46473667 | 0.00123287 |
| CDC25C | 0.77422974 | 0.27126502 | 3.14545308 | 4.4226E-07 | 2.43530333 | 0.00130513 |
| C5orf46 | 1.13325666 | 0.34883351 | 2.38719385 | 8.0531E-05 | 2.70530333 | 0.00139899 |
| KCNJ6 | 1.11006919 | 0.99002977 | 2.76239538 | 9.6553E-05 | 3.06645 | 0.00144935 |
| GINS1 | 1.22347386 | 0.38046447 | 2.04030923 | 0.00186591 | 2.496265 | 0.00158622 |
| CRYBG2 | 0.87982795 | 0.42336493 | 2.53855692 | 0.00170111 | 2.23349333 | 0.00163699 |
| PPP2R2C | 1.05732595 | 0.7293931 | 2.41556385 | 0.00030013 | 2.55403833 | 0.0017587 |
| MYBL2 | 0.95757277 | 0.8429739 | 2.22822231 | 0.01286456 | 2.133685 | 0.00201165 |
| EBF2 | 0.96632733 | 0.80095664 | 2.30179077 | 0.00745297 | 2.22428333 | 0.00239525 |
| B4GALNT4 | 1.24335247 | 0.20917048 | 1.05337923 | 0.58061599 | 1.30972167 | 0.00254544 |
| NXPH4 | 0.94644147 | 0.76776834 | 2.41203154 | 0.00350878 | 2.28284667 | 0.00329343 |
| CENPF | 1.04412691 | 0.98854534 | 2.20308308 | 0.00658887 | 2.30029833 | 0.00443792 |
| HOXA13 | 0.78497387 | 0.30292642 | 2.86614 | 1.952E-05 | 2.249845 | 0.00577499 |
| CACNG4 | 1.12896581 | 0.55405943 | 2.08590462 | 0.00904615 | 2.354915 | 0.00609705 |
| CENPA | 0.98227278 | 0.94630536 | 2.39298769 | 0.00852236 | 2.35056667 | 0.00761244 |
| HOXA10 | 0.87901953 | 0.4425548 | 2.37270231 | 0.00103784 | 2.08565167 | 0.010384 |
| E2F8 | 1.04828849 | 0.81023706 | 2.30148 | 0.00108915 | 2.412615 | 0.01102018 |
| CCNO | 0.80037841 | 0.3352244 | 2.65511077 | 0.00019295 | 2.12509333 | 0.01723908 |
| MAFA | 1.10325965 | 0.665832 | 1.06362692 | 0.40312763 | 1.17345667 | 0.01785996 |
| HS6ST2 | 0.72725138 | 0.2200008 | 2.65905077 | 3.7167E-05 | 1.93379833 | 0.01888521 |
| TOP2A | 0.96470821 | 0.90128788 | 2.02115 | 0.01381117 | 1.94982 | 0.02115081 |
| PRRX1 | 1.03276068 | 0.6640342 | 2.25223846 | 0.02141728 | 2.32602333 | 0.02547686 |
| SPINK5 | 1.0340706 | 0.38024536 | 1.06884385 | 0.33429036 | 1.10526 | 0.03141308 |

| | | | | | | |
|---|---|---|---|---|---|---|
| SLC22A31 | 1.02454345 | 0.73391712 | 1.15725692 | 0.01531797 | 1.18566 | 0.03426916 |
| NCAPG | 0.57719785 | 0.08295839 | 2.53900231 | 0.00018902 | 1.46550667 | 0.03476539 |
| ZBED2 | 0.80048409 | 0.5403893 | 2.70488615 | 0.00065064 | 2.16521833 | 0.03835975 |
| SLC2A1 | 0.79434238 | 0.47706038 | 2.82076308 | 0.00030008 | 2.24065167 | 0.04048174 |
| DEPDC1 | 0.71294939 | 0.17442679 | 2.81994769 | 7.643E-05 | 2.01048 | 0.041575 |
| NRCAM | 0.76998298 | 0.44103905 | 2.52459692 | 0.00099161 | 1.94389667 | 0.04371602 |
| C20orf204 | 1.06131875 | 0.5409399 | 1.12872154 | 0.14654624 | 1.19793333 | 0.05111414 |
| NOTUM | 1.03677415 | 0.64011742 | 1.14669462 | 0.10001068 | 1.18886333 | 0.0579736 |
| IGFBPL1 | 1.09474503 | 0.60474101 | 1.08609308 | 0.31044501 | 1.188995 | 0.08329436 |
| UBE2C | 0.76821389 | 0.25222404 | 1.82148923 | 0.0345552 | 1.39929333 | 0.12095767 |
| BPIFB2 | 1.20305392 | 0.11193887 | 0.95102692 | 0.39349666 | 1.14413667 | 0.12095804 |
| ZIC5 | 0.96690204 | 0.54986683 | 1.15998308 | 0.05072419 | 1.12159 | 0.20251785 |
| MAGEA10 | 0.91807455 | 0.21413943 | 1.15553615 | 0.02871957 | 1.06086833 | 0.22290894 |
| SIX4 | 0.96793062 | 0.86602113 | 1.12248231 | 0.06287446 | 1.086485 | 0.24794827 |
| ZIC2 | 1.00635073 | 0.83053268 | 1.09321231 | 0.2981986 | 1.100155 | 0.26538049 |
| AUNIP | 0.67776626 | 0.18864565 | 2.04648231 | 0.02099023 | 1.38703667 | 0.2839701 |
| TICRR | 0.8603772 | 0.21265855 | 1.08329231 | 0.23532104 | 0.93204 | 0.32514026 |
| KCNF1 | 1.05775139 | 0.94491268 | 1.02354077 | 0.79118716 | 1.08265167 | 0.3270386 |
| COL11A1 | 0.85172968 | 0.00453561 | 1.22612846 | 0.00159561 | 1.04433 | 0.33243582 |
| CCR8 | 1.03154589 | 0.88878498 | 1.04449385 | 0.52272134 | 1.07744333 | 0.37825244 |
| GPC3 | 0.99254625 | 0.95323082 | 1.07894385 | 0.39788712 | 1.07090167 | 0.41642794 |
| NPFFR2 | 0.87991288 | 0.39245284 | 1.06581385 | 0.48813422 | 0.93782333 | 0.42153137 |
| NAA11 | 0.99606062 | 0.94237537 | 1.06147154 | 0.47280677 | 1.05729 | 0.54634471 |
| PITX1 | 0.99874162 | 0.86291518 | 1.04040923 | 0.5804665 | 1.0391 | 0.59181755 |
| MEP1A | 0.99583584 | 0.96897681 | 1.03551538 | 0.63826195 | 1.03120333 | 0.62062074 |
| MAGEA12 | 0.94950841 | 0.82657897 | 1.06938846 | 0.4521716 | 1.01539333 | 0.84871221 |
| REG3A | 0.92636717 | 0.49295813 | 1.06071692 | 0.46714399 | 0.98261333 | 0.85992963 |
| KREMEN2 | 0.94434126 | 0.62830017 | 1.07225538 | 0.45595069 | 1.012575 | 0.91903906 |
| MAGEA6 | 0.93180212 | 0.68853294 | 1.08055846 | 0.30639057 | 1.00686667 | 0.93938049 |
| TRIM71 | 0.93833941 | 0.5899775 | 1.06918846 | 0.37938573 | 1.00326167 | 0.9557742 |
| SLC9A3 | 0.28829873 | 5.1639E-05 | 3.46554308 | 6.3312E-08 | 0.99911167 | 0.98265344 |

HCC, hepatocellular carcinoma; BM HCC, HCC with bone mestasis

**Table S6. $m^6A$ and transcript level of 68 genes in 6 HCC with BM (BM HCC), 13 HCC with metastasis (non-bone), and 10 advantage-stage HCC, as assessed using qRT-PCR analysis**

| Group | Metastasis(bone+) vs. Metastasis(bone-) | | Metastasis(bone-) vs. no-Metastasis | | Metastasis(bone+) vs. non-Metastasis | |
|---|---|---|---|---|---|---|
| **qRT-PCR** | **Foldchange** | **p-vaule** | **Foldchange** | **p-vaule** | **Foldchange** | **p-vaule** |
| ANLN | 4.45799924 | 0.00028511 | 0.98927077 | 0.94952835 | 4.41016833 | 0.00028633 |
| SFN | 1.10154115 | 0.4617522 | 2.67577385 | 0.0013056 | 2.947475 | 5.9502E-11 |
| C5orf46 | 1.58131456 | 0.02419206 | 2.19074692 | 0.00464888 | 3.46426 | 1.493E-06 |
| NEK2 | 1.29120705 | 0.49716443 | 1.97912231 | 0.03597505 | 2.55545667 | 4.799E-06 |
| NCAPG | 1.19007236 | 0.46015225 | 2.50851692 | 0.00043333 | 2.98531667 | 3.114E-05 |
| KCNJ6 | 1.29535481 | 0.38570583 | 2.31085077 | 0.00302023 | 2.99337167 | 6.2713E-05 |
| SLC22A11 | 1.16922359 | 0.57008517 | 2.85464077 | 0.00123025 | 3.33771333 | 6.6387E-05 |
| UBE2C | 1.16566487 | 0.49766389 | 2.68875308 | 0.00017063 | 3.134185 | 6.6712E-05 |
| CCNO | 1.08802888 | 0.99783159 | 2.47042615 | 0.00037513 | 2.687895 | 7.3908E-05 |
| MUC13 | 1.04485333 | 0.51786447 | 2.67675846 | 0.00024327 | 2.79682 | 0.00011042 |
| CDC25C | 1.46657546 | 0.22845257 | 1.77393077 | 0.04073805 | 2.60160333 | 0.0003606 |
| CENPF | 1.18166291 | 0.63527 | 2.59761615 | 0.0018171 | 3.06950667 | 0.00040994 |
| ZBED2 | 0.81766425 | 0.39103 | 2.65983462 | 0.00017317 | 2.17485167 | 0.00045124 |
| EBF2 | 1.07436872 | 0.41941612 | 2.59241538 | 0.00011239 | 2.78521 | 0.00060806 |
| NKD1 | 1.04666475 | 0.7655985 | 2.74194769 | 0.00015598 | 2.8699 | 0.00085506 |
| SLC2A1 | 0.93772885 | 0.47625525 | 2.18087385 | 0.01528809 | 2.04506833 | 0.00122641 |
| GP2 | 1.44549101 | 0.03349208 | 1.64506615 | 0.02728607 | 2.37792833 | 0.00128756 |
| PPP2R2C | 0.72196821 | 0.33792805 | 3.18877385 | 7.6745E-06 | 2.30219333 | 0.00146162 |
| NRCAM | 1.28862366 | 0.55791053 | 1.90137231 | 0.05087376 | 2.45015333 | 0.00157 |
| MYBL2 | 1.28100522 | 0.49816346 | 1.93432077 | 0.01827349 | 2.477875 | 0.0017065 |
| NXPH4 | 0.9881801 | 0.9593101 | 2.77047 | 0.00014769 | 2.73772333 | 0.00194947 |
| EXO1 | 1.04633886 | 0.64396244 | 2.03393 | 0.02172386 | 2.12818 | 0.00221534 |
| GABRQ | 1.11172141 | 0.50764558 | 1.11393615 | 0.2027629 | 1.23838667 | 0.00418726 |
| PRRX1 | 0.93340987 | 0.92050729 | 2.27227615 | 0.01050742 | 2.120965 | 0.00494784 |
| UHRF1 | 0.89660519 | 0.6440522 | 2.66035154 | 0.00021422 | 2.385285 | 0.00753053 |
| DEPDC1 | 1.02444015 | 0.95484424 | 2.29797385 | 0.00306156 | 2.35413667 | 0.00825405 |
| HOXA13 | 0.87980904 | 0.30132882 | 2.54984308 | 0.00245012 | 2.243375 | 0.01027168 |
| CACNG4 | 1.07858113 | 0.93869853 | 2.2095 | 0.01384237 | 2.383125 | 0.0122228 |
| MELK | 0.95055752 | 0.85293505 | 2.40184308 | 0.00160634 | 2.28309 | 0.01843884 |
| CRYBG2 | 0.82676577 | 0.63201474 | 2.68936308 | 0.00018324 | 2.22347333 | 0.02038293 |
| SPINK5 | 1.07403202 | 0.49103061 | 1.13720538 | 0.10852529 | 1.221395 | 0.02224495 |
| TICRR | 1.07629248 | 0.26185231 | 1.07953308 | 0.26456885 | 1.16189333 | 0.03148091 |
| HS6ST2 | 0.96788771 | 0.8376501 | 2.32679538 | 0.00398118 | 2.25207667 | 0.03510196 |
| TRIM71 | 0.880229 | 0.03239603 | 1.02298 | 0.72272453 | 0.90045667 | 0.03601479 |
| IGFBPL1 | 1.04727816 | 0.8043438 | 1.10446462 | 0.2005266 | 1.15668167 | 0.03633978 |
| CA12 | 1.09524425 | 0.46186077 | 1.08101154 | 0.35716485 | 1.18397167 | 0.03884897 |
| PITX1 | 1.09526409 | 0.32149353 | 1.09367231 | 0.28610328 | 1.19786 | 0.04605858 |

| | | | | | | |
|---|---|---|---|---|---|---|
| REG3A | 1.18678385 | 0.04642721 | 0.98158846 | 0.82133396 | 1.16493333 | 0.04740807 |
| HOXA10 | 0.58384835 | 0.02835469 | 3.08751692 | 5.6223E-06 | 1.80264167 | 0.05224957 |
| E2F8 | 0.74579361 | 0.32899377 | 2.67438154 | 7.3488E-05 | 1.99453667 | 0.05563304 |
| KIF15 | 0.64892629 | 0.180331 | 2.43578769 | 0.00087718 | 1.58064667 | 0.05656971 |
| AUNIP | 0.68693738 | 0.11407983 | 2.44790385 | 0.00028648 | 1.68155667 | 0.05962061 |
| GINS1 | 0.84682113 | 0.55608483 | 2.16485308 | 0.00353303 | 1.83324333 | 0.08470484 |
| COL11A1 | 1.1182519 | 0.5022626 | 1.02774846 | 0.7543766 | 1.14928167 | 0.09136707 |
| TOP2A | 0.67236628 | 0.30713331 | 2.90163769 | 9.1905E-05 | 1.95096333 | 0.10130106 |
| SLC9A3 | 0.30749919 | 0.00010015 | 3.44191154 | 1.5428E-07 | 1.058385 | 0.10254363 |
| ZIC5 | 1.05339675 | 0.74189302 | 1.11368769 | 0.26848925 | 1.173155 | 0.10517722 |
| CENPA | 0.75467941 | 0.49912776 | 2.31328692 | 0.01497755 | 1.74579 | 0.10571529 |
| NAA11 | 0.99482784 | 0.92524126 | 1.16489 | 0.06156614 | 1.158865 | 0.13337587 |
| B4GALNT4 | 1.02028452 | 0.74460195 | 1.09386154 | 0.27335038 | 1.11605 | 0.15507576 |
| OTX1 | 0.73176816 | 0.48516841 | 1.95621385 | 0.0223581 | 1.431495 | 0.20228238 |
| SIX4 | 1.02865692 | 0.62260386 | 1.11705692 | 0.15003839 | 1.14906833 | 0.2061045 |
| PRAME | 0.98358445 | 0.61182817 | 1.13489154 | 0.09945979 | 1.11626167 | 0.20626853 |
| NOTUM | 0.81844445 | 0.06743796 | 1.14501154 | 0.11507842 | 0.93712833 | 0.27178292 |
| KCNF1 | 0.81345097 | 0.1271357 | 1.14072231 | 0.12340461 | 0.92792167 | 0.35368213 |
| CCR8 | 0.98500473 | 0.4932481 | 1.07151769 | 0.34671661 | 1.05545 | 0.35916626 |
| NPFFR2 | 0.98496154 | 0.85254367 | 1.08016231 | 0.38714971 | 1.06391833 | 0.3796466 |
| C20orf204 | 0.97880671 | 0.90123202 | 1.08479538 | 0.27217329 | 1.061805 | 0.41201987 |
| MAGEA6 | 0.97845846 | 0.88054508 | 1.09126077 | 0.32578749 | 1.06775333 | 0.42457349 |
| MAGEA12 | 0.99828898 | 0.81690634 | 1.07200923 | 0.37366828 | 1.070175 | 0.42652201 |
| SLC22A31 | 0.92642259 | 0.98877706 | 1.14292154 | 0.14057421 | 1.05882833 | 0.45139647 |
| MAGEA10 | 1.03655862 | 0.62234112 | 1.02439615 | 0.78765034 | 1.06184667 | 0.5448025 |
| MEP1A | 0.92529375 | 0.35763923 | 1.13531154 | 0.14169459 | 1.05049667 | 0.62052395 |
| MAFA | 1.0265657 | 0.57644566 | 1.00631 | 0.93239014 | 1.03304333 | 0.62428241 |
| KREMEN2 | 0.88032697 | 0.5379879 | 1.12120462 | 0.11246363 | 0.98702667 | 0.87954951 |
| GPC3 | 0.94608064 | 0.62293466 | 1.06059846 | 0.41938799 | 1.00341167 | 0.96698655 |
| BPIFB2 | 0.85927347 | 0.06434619 | 1.16607231 | 0.04403666 | 1.001975 | 0.97407384 |
| ZIC2 | 1.0742991 | 0.67686375 | 0.93348615 | 0.15569744 | 1.00284333 | 0.97470409 |

HCC, hepatocellular carcinoma; BM HCC, HCC with bone mestasis

**Table S7. The expression of 238 genes in HCC and normal tissues and Correlation coefficient(R) with ANLN expression in HCC based on GEPIA data.**

| Gene Name | UP/Down (LIHC/Normal) | Fold change （> 2/＜0.5） | p-Value | DFS- p-Value | OS- p-Value | R | p-Value |
|---|---|---|---|---|---|---|---|
| KIF2C | UP | YES | YES | 6.20E-06 | 1.10E-05 | 0.86 | 0 |
| CENPA | UP | YES | YES | 0.0047 | 0.00078 | 0.86 | 0 |
| PRR11 | UP | YES | YES | 0.011 | 0.0063 | 0.85 | 0 |
| GTSE1 | NO | NO | NO | 0.0059 | 0.028 | 0.85 | 0 |
| DLGAP5 | UP | YES | YES | 0.0033 | 0.00039 | 0.82 | 0 |
| NCAPH | UP | YES | YES | 0.00019 | 0.02 | 0.78 | 0 |
| PHF19 | UP | YES | YES | 6.80E-05 | 0.0025 | 0.76 | 0 |
| MYBL2 | UP | YES | YES | 0.0047 | 0.0034 | 0.76 | 0 |
| BIRC5 | UP | YES | YES | 0.002 | 6.70E-05 | 0.76 | 0 |
| CDCA3 | UP | YES | YES | 0.0017 | 0.00071 | 0.76 | 0 |
| STIL | NO | NO | NO | 0.0069 | 0.013 | 0.75 | 0 |
| CDCA5 | UP | YES | YES | 0.00014 | 0.00021 | 0.75 | 0 |
| HMGB2 | UP | YES | YES | 0.00044 | 0.0039 | 0.69 | 0 |
| TACC3 | UP | YES | YES | 0.00021 | 0.0028 | 0.67 | 0 |
| PIF1 | NO | NO | NO | 0.02 | 0.005 | 0.67 | 0 |
| CBX5 | NO | NO | NO | 0.077 | 0.037 | 0.65 | 0 |
| FAM83D | UP | YES | YES | 0.011 | 0.0042 | 0.64 | 0 |
| EME1 | NO | NO | NO | 7.80E-05 | 0.0029 | 0.63 | 0 |
| IQGAP3 | UP | YES | YES | 0.058 | 0.00064 | 0.62 | 0 |
| CNTNAP1 | NO | NO | NO | 0.24 | 0.0017 | 0.62 | 0 |
| SAPCD2 | NO | NO | NO | 0.14 | 0.017 | 0.61 | 0 |
| AURKA | UP | YES | YES | 0.0012 | 0.00022 | 0.59 | 0 |
| ARHGAP33 | NO | NO | NO | 0.0028 | 0.17 | 0.57 | 0 |
| TRIM65 | NO | NO | NO | 0.0032 | 0.014 | 0.56 | 0 |
| CARD19 | NO | NO | NO | 0.0047 | 0.034 | 0.49 | 0 |
| ARHGAP22 | NO | NO | NO | 0.27 | 0.041 | 0.49 | 0 |
| UAP1L1 | NO | NO | NO | 0.65 | 0.045 | 0.47 | 0 |
| SH3BP1 | NO | NO | NO | 0.81 | 0.81 | 0.47 | 0 |
| PAQR4 | UP | YES | YES | 0.0089 | 0.0088 | 0.47 | 0 |
| PIK3R2 | UP | YES | YES | 0.16 | 0.094 | 0.46 | 0 |
| RMI2 | UP | YES | YES | 0.0021 | 0.00082 | 0.44 | 0 |
| LRRK1 | NO | NO | NO | 0.22 | 0.96 | 0.44 | 0 |
| ITPKA | UP | YES | YES | 0.094 | 0.41 | 0.44 | 0 |
| PKMYT1 | UP | YES | YES | 0.0037 | 0.0017 | 0.42 | 0 |
| IFI30 | UP | YES | YES | 0.58 | 0.15 | 0.42 | 0 |
| TRIM47 | NO | NO | NO | 0.051 | 0.39 | 0.41 | 0 |
| PLAU | UP | YES | YES | 0.7 | 0.18 | 0.41 | 4.40E-16 |
| FBLN1 | UP | YES | YES | 0.76 | 0.83 | 0.41 | 4.40E-16 |
| PKN3 | NO | NO | NO | 0.014 | 0.1 | 0.4 | 1.30E-15 |

| | | | | | | | |
|---|---|---|---|---|---|---|---|
| NLRC5 | NO | NO | NO | 1 | 0.87 | 0.4 | 8.90E-16 |
| SYNGR2 | UP | YES | YES | 0.0025 | 0.053 | 0.38 | 7.00E-14 |
| RAP1GAP | NO | NO | NO | 0.59 | 0.014 | 0.38 | 5.30E-14 |
| CHRM4 | NO | NO | NO | 0.43 | 0.35 | 0.38 | 7.20E-14 |
| ZNF580 | NO | NO | NO | 0.0086 | 0.0012 | 0.37 | 2.70E-03 |
| ZNF514 | NO | NO | NO | 0.008 | 0.15 | 0.37 | 3.50E-13 |
| NSMF | NO | NO | NO | 0.035 | 0.33 | 0.37 | 2.40E-13 |
| MGAT5B | NO | NO | NO | 0.6 | 0.027 | 0.37 | 2.50E-13 |
| CAPG | UP | YES | YES | 0.28 | 0.00045 | 0.37 | 4.40E-13 |
| PLB1 | NO | NO | NO | 0.6 | 0.78 | 0.36 | 1.50E-12 |
| PDZD7 | NO | NO | NO | 0.63 | 0.14 | 0.36 | 6.00E-13 |
| UNC5B | NO | NO | NO | 0.09 | 0.018 | 0.34 | 1.30E-11 |
| TOX2 | NO | NO | NO | 0.67 | 0.76 | 0.32 | 4.70E-10 |
| ENTPD2 | NO | NO | NO | 0.39 | 0.85 | 0.32 | 3.60E-10 |
| ADM2 | UP | YES | YES | 0.87 | 0.11 | 0.32 | 1.90E-10 |
| TLDC2 | NO | NO | NO | 0.086 | 0.28 | 0.31 | 1.10E-09 |
| SYNC | NO | NO | NO | 0.093 | 0.96 | 0.31 | 1.00E-09 |
| RINL | NO | NO | NO | 0.63 | 0.58 | 0.31 | 9.70E-10 |
| OPRL1 | NO | NO | NO | 0.7 | 0.0025 | 0.31 | 1.00E-09 |
| ITGB4 | UP | YES | YES | 0.35 | 0.97 | 0.3 | 3.70E-08 |
| FOXRED2 | UP | YES | YES | 0.45 | 0.1 | 0.3 | 5.60E-09 |
| CARD14 | NO | NO | NO | 0.19 | 0.14 | 0.3 | 5.70E-09 |
| BAIAP2L2 | NO | NO | NO | 0.17 | 0.03 | 0.3 | 3.60E-09 |
| MDK | UP | YES | YES | 0.18 | 0.19 | 0.3 | 2.70E-09 |
| ARHGEF17 | NO | NO | NO | 0.58 | 0.24 | 0.29 | 8.00E-09 |
| MAPK12 | NO | NO | NO | 0.48 | 0.027 | 0.28 | 5.10E-08 |
| ERBB3 | NO | NO | NO | 0.0016 | 0.0058 | 0.28 | 6.80E-08 |
| AGRN | NO | NO | NO | 0.011 | 0.003 | 0.28 | 5.90E-08 |
| TAPBP | NO | NO | NO | 0.5 | 0.38 | 0.27 | 1.60E-07 |
| SLC6A9 | NO | NO | NO | 0.31 | 0.58 | 0.27 | 1.40E-07 |
| HVCN1 | NO | NO | NO | 0.16 | 0.81 | 0.27 | 1.80E-07 |
| GRHPR | NO | NO | NO | 0.74 | 0.31 | 0.27 | 1.30E-07 |
| GNG2 | NO | NO | NO | 0.54 | 0.54 | 0.27 | 1.30E-07 |
| ADGRG1 | NO | NO | NO | 0.68 | 0.2 | 0.27 | 1.60E-07 |
| MCAM | UP | YES | YES | 0.19 | 0.062 | 0.26 | 3.20E-07 |
| MAPK8IP2 | NO | NO | NO | 0.47 | 0.45 | 0.26 | 4.5-07 |
| IQCK | NO | NO | NO | 0.8 | 0.57 | 0.26 | 2.80E-07 |
| ADORA2A | NO | NO | NO | 0.8 | 0.84 | 0.26 | 6.3-07 |
| TMC8 | NO | NO | NO | 0.032 | 0.36 | 0.25 | 1.00E-06 |
| SYNGR4 | NO | NO | NO | 0.17 | 0.012 | 0.25 | 1.40E-06 |
| CD8A | NO | NO | NO | 0.087 | 0.079 | 0.25 | 1.40E-06 |
| AHNAK2 | NO | NO | NO | 1 | 0.51 | 0.25 | 1.30E-06 |
| WNK2 | NO | NO | NO | 0.84 | 0.32 | 0.24 | 4.50E-06 |
| VIL1 | UP | YES | YES | 0.85 | 0.7 | 0.24 | 3.50E-06 |

| | | | | | | | |
|---|---|---|---|---|---|---|---|
| TTLL6 | NO | NO | NO | 0.041 | 0.23 | 0.24 | 3.80E-06 |
| SLC44A2 | NO | NO | NO | 0.53 | 0.93 | 0.24 | 4.20E-06 |
| RNF224 | NO | NO | NO | 0.89 | 0.94 | 0.24 | 1.90E-06 |
| RNF223 | NO | NO | NO | / | / | 0.24 | 2.40E-06 |
| ACSS1 | NO | NO | NO | 0.7 | 0.084 | 0.23 | 1.10E-05 |
| ZC3H6 | NO | NO | NO | 0.73 | 0.82 | 0.22 | 1.60E-05 |
| TRIM7 | NO | NO | NO | 0.31 | 0.059 | 0.22 | 1.70E-05 |
| STMN3 | NO | NO | NO | 0.23 | 0.83 | 0.22 | 2.10E-05 |
| LLGL2 | NO | NO | NO | 0.13 | 0.7 | 0.22 | 1.5-05 |
| CLIC3 | NO | NO | NO | 0.38 | 0.35 | 0.22 | 2.70E-05 |
| QRICH2 | NO | NO | NO | 0.096 | 0.55 | 0.21 | 4.30E-05 |
| MST1R | NO | NO | NO | 0.026 | 0.038 | 0.21 | 6.20E-05 |
| MN1 | NO | NO | NO | 0.61 | 0.82 | 0.21 | 5.20E-05 |
| MAPK11 | NO | NO | NO | 0.49 | 0.72 | 0.21 | 7.10E-05 |
| ABCA2 | NO | NO | NO | 0.026 | 0.14 | 0.21 | 4.10E-05 |
| SPON2 | UP | YES | YES | 0.77 | 0.027 | 0.2 | 0.00014 |
| MUC5B | NO | NO | NO | 0.42 | 0.45 | 0.2 | 0.00012 |
| MCF2L | NO | NO | NO | 0.65 | 0.18 | 0.2 | 0.00011 |
| FGFR3 | NO | NO | NO | 0.33 | 0.94 | 0.2 | 8.30E-05 |
| ZNF385C | NO | NO | NO | 0.21 | 0.4 | 0.19 | 0.00032 |
| ZNF219 | NO | NO | NO | 0.12 | 0.74 | 0.19 | 0.00027 |
| PRPH | NO | NO | NO | 0.56 | 0.45 | 0.19 | 0.00028 |
| PCDH1 | NO | NO | NO | 0.18 | 0.05 | 0.19 | 0.00029 |
| HR | NO | NO | NO | 0.64 | 0.28 | 0.19 | 0.00027 |
| HES4 | NO | NO | NO | 0.41 | 0.034 | 0.19 | 0.00021 |
| DOK7 | NO | NO | NO | 0.55 | 0.2 | 0.19 | 0.00028 |
| STAT6 | NO | NO | NO | 0.97 | 0.66 | 0.18 | 0.00055 |
| SH2D6 | NO | NO | NO | 0.75 | 0.03 | 0.18 | 0.00037 |
| RAB3D | NO | NO | NO | 0.79 | 0.44 | 0.18 | 0.00036 |
| JAG2 | NO | NO | NO | 0.77 | 0.89 | 0.18 | 0.00037 |
| ETV4 | UP | YES | YES | 0.22 | 0.0024 | 0.18 | 6.00E-04 |
| CRIP1 | UP | YES | YES | 0.58 | 0.56 | 0.18 | 0.00038 |
| ACE | NO | NO | NO | 0.94 | 0.72 | 0.18 | 0.00051 |
| RASAL1 | NO | NO | NO | 0.71 | 0.42 | 0.17 | 0.0014 |
| PITPNM3 | NO | NO | NO | 0.22 | 0.41 | 0.17 | 0.0012 |
| VWA7 | NO | NO | NO | 0.069 | 0.13 | 0.16 | 0.0016 |
| NTSR1 | NO | NO | NO | 0.68 | 0.21 | 0.16 | 0.0017 |
| NEURL3 | NO | NO | NO | 0.029 | 0.28 | 0.16 | 0.0018 |
| GDPD3 | NO | NO | NO | 0.51 | 0.2 | 0.16 | 0.002 |
| BEST4 | NO | NO | NO | 0.038 | 0.78 | 0.16 | 0.0027 |
| PPL | NO | NO | NO | 0.71 | 0.24 | 0.15 | 0.0048 |
| PIGZ | NO | NO | NO | 0.058 | 0.017 | 0.15 | 0.0032 |
| KLHDC8B | NO | NO | NO | 0.22 | 0.014 | 0.15 | 0.003 |
| INHBE | NO | NO | NO | 0.91 | 0.53 | 0.14 | 0.0055 |

| | | | | | | | |
|---|---|---|---|---|---|---|---|
| FSCN2 | NO | NO | NO | 0.088 | 0.38 | 0.14 | 0.00084 |
| FAM83E | NO | NO | NO | 0.71 | 0.65 | 0.14 | 0.0057 |
| AIFM3 | NO | NO | NO | 0.37 | 0.49 | 0.14 | 0.0084 |
| ADAP1 | NO | NO | NO | 0.59 | 0.45 | 0.14 | 0.0063 |
| PSCA | NO | NO | NO | 0.16 | 0.035 | 0.13 | 0.014 |
| KCNQ1 | NO | NO | NO | 0.83 | 0.12 | 0.13 | 0.011 |
| CAPS | NO | NO | NO | 0.13 | 0.6 | 0.13 | 0.014 |
| VGF | NO | NO | NO | 0.89 | 0.27 | 0.12 | 0.018 |
| SEMA3B | NO | NO | NO | 0.38 | 0.21 | 0.12 | 0.023 |
| RAC3 | UP | YES | YES | 0.026 | 0.15 | 0.11 | 0.03 |
| PECAM1 | UP | YES | YES | 0.66 | 0.77 | 0.11 | 0.043 |
| LBX1 | NO | NO | NO | / | / | 0.11 | 0.032 |
| HSD3B7 | NO | NO | NO | 0.38 | 0.37 | 0.11 | 0.04 |
| GSTP1 | NO | NO | NO | 0.029 | 0.97 | 0.11 | 0.041 |
| SPOCK2 | NO | NO | NO | 0.094 | 0.17 | 0.1 | 0.052 |
| GDF15 | UP | YES | YES | 0.6 | 0.072 | 0.1 | 0.046 |
| FAM20C | NO | NO | NO | 0.31 | 0.84 | 0.1 | 0.044 |
| AIF1L | NO | NO | NO | 0.069 | 0.25 | 0.1 | 0.049 |
| ISYNA1 | NO | NO | NO | 0.56 | 0.41 | 0.1 | 0.051 |
| SYT17 | NO | NO | NO | 0.73 | 0.15 | 0.099 | 0.058 |
| BRSK2 | NO | NO | NO | 0.2 | 0.033 | 0.098 | 0.06 |
| EFNA2 | NO | NO | NO | 0.15 | 0.72 | 0.097 | 0.063 |
| NPDC1 | NO | NO | NO | 0.16 | 0.79 | 0.092 | 0.078 |
| ADSSL1 | NO | NO | NO | 0.0042 | 0.025 | 0.092 | 0.076 |
| ARHGEF16 | NO | NO | NO | 0.12 | 0.021 | 0.091 | 0.082 |
| SSTR5 | NO | NO | NO | / | / | 0.086 | 0.1 |
| BCAS3 | NO | NO | NO | 0.87 | 0.97 | 0.082 | 0.11 |
| TJP3 | NO | NO | NO | 0.67 | 0.48 | 0.078 | 0.13 |
| LRRC75B | NO | NO | NO | 0.099 | 0.87 | 0.077 | 0.14 |
| FAS | NO | NO | NO | 0.72 | 0.25 | 0.076 | 0.15 |
| S100A14 | NO | NO | NO | 0.58 | 0.26 | 0.073 | 0.16 |
| RAB37 | NO | NO | NO | 0.13 | 0.97 | 0.068 | 0.19 |
| TLX1 | NO | NO | NO | 0.013 | 0.68 | 0.067 | 0.2 |
| MYOM3 | NO | NO | NO | 0.96 | 0.47 | 0.063 | 0.23 |
| ACTL8 | NO | NO | NO | 0.85 | 0.071 | 0.061 | 0.25 |
| NPB | NO | NO | NO | 0.72 | 0.18 | 0.06 | 0.25 |
| MUC5AC | NO | NO | NO | / | / | 0.056 | 0.28 |
| MGAT4B | NO | NO | NO | 0.048 | 0.0045 | 0.056 | 0.29 |
| CRAT | NO | NO | NO | 0.66 | 0.24 | 0.056 | 0.28 |
| FKBP5 | NO | NO | NO | 0.77 | 0.92 | 0.055 | 0.29 |
| NGEF | NO | NO | NO | 0.65 | 0.76 | 0.054 | 0.3 |
| IGF2 | DOWN | YES | YES | 0.29 | 0.041 | 0.051 | 0.33 |
| GIP | NO | NO | NO | / | / | 0.049 | 0.35 |
| SNCG | UP | YES | YES | 0.5 | 0.21 | 0.048 | 0.36 |

| | | | | | | | | |
|---|---|---|---|---|---|---|---|---|
| FN3K | UP | YES | YES | 0.92 | 0.57 | 0.047 | 0.37 |
| PDLIM2 | NO | NO | NO | 0.42 | 0.81 | 0.045 | 0.39 |
| PRSS36 | NO | NO | NO | 0.79 | 0.82 | 0.041 | 0.44 |
| PALM | NO | NO | NO | 0.94 | 0.24 | 0.036 | 0.49 |
| EGFL7 | NO | NO | NO | 0.24 | 0.77 | 0.035 | 0.51 |
| GGT1 | NO | NO | NO | 0.15 | 0.049 | 0.034 | 0.51 |
| OTOF | NO | NO | NO | 0.56 | 0.16 | 0.032 | 0.54 |
| CST3 | NO | NO | NO | 0.58 | 0.12 | 0.032 | 0.54 |
| SLC2A10 | NO | NO | NO | 0.78 | 0.93 | 0.027 | 0.61 |
| GLI2 | NO | NO | NO | 0.51 | 0.38 | 0.024 | 0.65 |
| SCNN1D | NO | NO | NO | 0.077 | 0.58 | 0.023 | 0.66 |
| ITGA7 | NO | NO | NO | 0.43 | 0.15 | 0.018 | 0.73 |
| COMTD1 | NO | NO | NO | 0.87 | 0.4 | 0.018 | 0.74 |
| DBNDD1 | UP | YES | YES | 0.86 | 0.32 | 0.017 | 0.74 |
| TMPRSS9 | NO | NO | NO | 0.37 | 0.88 | 0.014 | 0.78 |
| TMEM105 | NO | NO | NO | 0.58 | 1 | 0.013 | 0.8 |
| RGS11 | NO | NO | NO | 0.18 | 0.18 | 0.013 | 0.81 |
| FBXL16 | NO | NO | NO | 0.89 | 0.84 | 0.0082 | 0.88 |
| CHDH | NO | NO | NO | 0.72 | 0.61 | 0.0016 | 0.98 |
| SEMA4G | NO | NO | NO | 0.94 | 0.5 | 0.00092 | 0.99 |
| PTGDS | NO | NO | NO | 0.089 | 0.67 | -0.0051 | 0.92 |
| ALPI | NO | NO | NO | 0.47 | 0.23 | -0.0071 | 0.89 |
| SPTBN2 | DOWN | YES | YES | 0.72 | 0.24 | -0.0086 | 0.87 |
| PANX2 | NO | NO | NO | 0.81 | 0.26 | -0.013 | 0.8 |
| TSPAN10 | NO | NO | NO | 0.81 | 0.99 | -0.013 | 0.8 |
| TPSG1 | NO | NO | NO | 0.57 | 0.25 | -0.014 | 0.79 |
| CPLX1 | NO | NO | NO | 0.21 | 0.16 | -0.016 | 0.76 |
| ADAMTSL2 | DOWN | YES | YES | 0.0026 | 0.063 | -0.016 | 0.75 |
| KLHDC2 | NO | NO | NO | 0.79 | 0.52 | -0.018 | 0.73 |
| SLC25A10 | NO | NO | NO | 0.86 | 0.54 | -0.027 | 0.6 |
| NPW | NO | NO | NO | 0.55 | 0.68 | -0.035 | 0.51 |
| CDHR2 | DOWN | YES | YES | 0.38 | 0.26 | -0.035 | 0.51 |
| AMN | DOWN | YES | YES | 0.52 | 0.93 | -0.035 | 0.5 |
| MYO7A | DOWN | YES | YES | 0.43 | 0.89 | -0.036 | 0.49 |
| SLC22A18AS | NO | NO | NO | 0.62 | 0.38 | -0.072 | 0.17 |
| RAB26 | NO | NO | NO | 0.73 | 0.42 | -0.083 | 0.11 |
| SDC1 | NO | NO | NO | 0.64 | 0.54 | -0.086 | 0.099 |
| PNPLA7 | DOWN | YES | YES | 0.61 | 0.15 | -0.091 | 0.081 |
| NR0B2 | NO | NO | NO | 0.37 | 0.21 | -0.094 | 0.072 |
| EEF1A2 | UP | YES | YES | 0.011 | 0.56 | -0.097 | 0.062 |
| PLCH2 | DOWN | YES | YES | 0.35 | 0.69 | -0.1 | 0.048 |
| TNXB | NO | NO | NO | 0.046 | 0.58 | -0.12 | 0.027 |
| MLXIPL | DOWN | YES | YES | 0.98 | 0.023 | -0.12 | 0.017 |
| NDRG2 | DOWN | YES | YES | 0.14 | 0.064 | -0.13 | 0.014 |

| | | | | | | | |
|---|---|---|---|---|---|---|---|
| NUDT8 | NO | NO | NO | 0.59 | 0.76 | -0.14 | 0.0066 |
| SLC22A18 | NO | NO | NO | 0.3 | 0.57 | -0.18 | 0.00052 |
| REEP6 | NO | NO | NO | 0.4 | 0.91 | -0.18 | 0.00071 |
| GCGR | DOWN | YES | YES | 0.15 | 0.044 | -0.18 | 0.00046 |
| F7 | NO | NO | NO | 0.0086 | 0.14 | -0.18 | 0.00043 |
| RARRES2 | NO | NO | NO | 0.8 | 0.9 | -0.2 | 0.00011 |
| LIMS2 | NO | NO | NO | 0.034 | 0.0048 | -0.2 | 7.50E-05 |
| GLTPD2 | NO | NO | NO | 0.015 | 0.22 | -0.2 | 0.00012 |
| FBP1 | DOWN | YES | YES | 0.013 | 0.12 | -0.21 | 4.40E-05 |
| COL18A1 | NO | NO | NO | 0.079 | 0.006 | -0.26 | 3.30E-07 |
| GAMT | NO | NO | NO | 0.13 | 0.23 | -0.27 | 2.30E-07 |
| C8G | NO | NO | NO | 0.7 | 0.49 | -0.29 | 9.00E-09 |
| BTD | NO | NO | NO | 0.85 | 0.28 | -0.15 | 0.0045 |
| TNNC2 | NO | NO | NO | 0.37 | 0.72 | -0.085 | 0.1 |
| PKD2L1 | NO | NO | NO | 0.3 | 0.47 | -0.011 | 0.84 |
| TOGARAM2 | / | / | / | / | / | / | / |
| SNORC | / | / | / | / | / | / | / |
| PLPPR2 | / | / | / | / | / | / | / |
| LOC105373989 | / | / | / | / | / | / | / |
| CBARP | / | / | / | / | / | / | / |
| CATSPERZ | / | / | / | / | / | / | / |
| C10orf143 | / | / | / | / | / | / | / |
| ATP5F1D | / | / | / | / | / | / | / |

HCC, hepatocellular carcinoma; GEPIA, gene expression profile interactive analysis

**Table S8. Correlation analysis of *KIF2C, MYBL2, HMGB2, AURKA* expression and Nucleus, Cytoplasm *ANLN* in HCC, respectively.**

| Substance | | Nucleus *ANLN* | | P value# | Cytoplasm *ANLN* | | P value# |
|---|---|---|---|---|---|---|---|
| | | High(N=132) | Low(N=133) | | High(N=132) | Low(N=133) | |
| *KIF2C* | High(N=132) | 85 | 47 | <0.001 | 59 | 73 | 0.097 |
| | Low(N=133) | 57 | 88 | | 73 | 60 | |
| *MYBL2* | High(N=132) | 81 | 50 | <0.001 | 62 | 69 | 0.424 |
| | Low(N=133) | 51 | 83 | | 70 | 64 | |
| *HMGB2* | High(N=132) | 77 | 56 | <0.001 | 64 | 69 | 0.581 |
| | Low(N=133) | 55 | 77 | | 68 | 64 | |
| *AURKA* | High(N=132) | 75 | 57 | <0.001 | 60 | 72 | 0.158 |
| | Low(N=133) | 57 | 76 | | 72 | 61 | |

#Note：the $\chi 2$ test was used for comparison between groups.

**Table S9. Antibodies and dilutions used in this study.**

| Cat. No | Antibody | Species | Dilution | Company | Application |
|---|---|---|---|---|---|
| ab195352 | Mettl3 | Rabbit | 1:1000 | Abcam | WB |
| ab220162 | YTHDF1 | Rabbit | 1:1000 | Abcam | WB |
| A6524 | ANLN | Rabbit | 1:1000 | Abclonal | WB |
| ab231778 | SP1 | Rabbit | 1:1000 | Abcam | WB |
| ab215206 | FOXP3 | Rabbit | 1:1000 | Abcam | WB |
| AP0115 | S2448P-mTOR | Rabbit | 1:1000 | Abclonal | WB |
| A2445 | mTOR | Rabbit | 1:1000 | Abclonal | WB |
| ab32572 | β-Catentin | Rabbit | 1:1000 | Abcam | WB |
| AF5131 | VEGFA | Rabbit | 1:1000 | Affinity | WB |
| AF6294 | STAT3 | Rabbit | 1:1000 | Affinity | WB |
| AF3293 | Phospho (Tyr705)-STAT3 | Rabbit | 1:1000 | Affinity | WB |
| AF6432 | 4E-BP1 | Rabbit | 1:1000 | Affinity | WB |
| AF3432 | Phospho-4E-BP1 (Thr45) | Rabbit | 1:1000 | Affinity | WB |
| AF6226 | S6K1 | Rabbit | 1:1000 | Affinity | WB |
| AF3228 | p70 (Thr389/Thr412)-S6K1 | Rabbit | 1:1000 | Affinity | WB |
| A16909 | Lamin B1 | Rabbit | 1:1000 | Abclonal | WB |
| ab52866 | α-Tubulin | Rabbit | 1:1000 | Abcam | WB |
| A5449 | KIF2C | Rabbit | 1:1000 | Abclonal | WB |
| MABE886 | MYBL2 | Mouse | 1 μg/mL | Sigma-Aldrich | WB |
| A9168 | HMGB2 | Rabbit | 1:1000 | Abclonal | WB |
| A15728 | AURKA | Rabbit | 1:1000 | Abclonal | WB |
| ab8226 | ACTIN | Mouse | 1:10000 | Abclonal | WB |
| ab6721 | Goat Anti-Rabbit IgG H&L (HRP) | Goat | 1:10000 | Abcam | WB |
| ab6789 | Goat Anti-Mouse IgG H&L (HRP) | Goat | 1:10000 | Abcam | WB |
| DF12020 | Mettl3 | Rabbit | 1:50 | Affinity | IHC |
| 17479-1-AP | YTHDF1 | Rabbit | 1:50 | proteintech | IHC |
| DF13590 | ANLN | Rabbit | 1:50 | Affinity | IHC |
| A5449 | KIF2C | Rabbit | 1:50 | Abclonal | IHC |
| DF7817 | MYBL2 | Rabbit | 1:50 | Affinity | IHC |
| A9168 | HMGB2 | Rabbit | 1:50 | Abclonal | IHC |
| A15728 | AURKA | Rabbit | 1:50 | Abclonal | IHC |
| A17924 | N6-methyladenosine / m6A Rabbit pAb | Rabbit | 1:50 | Abclonal | IP, RIP, and ChIP |
| AE063 | anti-FLAG | Rabbit | 1:50 | Abclonal | IP, RIP, and ChIP |
| A6524 | ANLN | Rabbit | 1:50 | Abclonal | IP, RIP, and ChIP |
| ab215206 | FOXP3 | Rabbit | 1:30 | Abcam | IP, RIP, and ChIP |
| ab231778 | SP1 | Rabbit | 1:30 | Abcam | IP, RIP, and ChIP |
| AE012 | anti-GFP | Mouse | 1:50 | Abclonal | IP, RIP, and ChIP |
| ab205718 | Goat Anti-Rabbit IgG H&L (HRP) | Goat | 1:50 | Abcam | IP, RIP, and ChIP |
| ab6789 | Goat Anti-Mouse IgG H&L (HRP) | Goat | 1:50 | Abcam | IP, RIP, and ChIP |
| ab32572 | β-Catentin | Rabbit | 1:50 | Abcam | IF |
| ab7090 | Goat Anti-Rabbit IgG H&L (HRP) | Goat | 1:50 | Abcam | IF |

WB, western blot; IHC, immunohistochemistry

**Table S10. Primer sequences for plasmid construction**

| Gene Name | sequence (5'-3') |
|---|---|
| Mettl3-KD#1 | GCAAGAATTCTGTGACTATGG |
| Mettl3-KD#1 | GCTGCACTTCAGACGAATTAT |
| Mettl3-KD#1 | GCTACCTGGACGTCAGTATCT |
| YTHDF1-KD#1 | GGATACAGTTCATGACAATGA |
| YTHDF1-KD#2 | GCACTGACTGGTGTCCTTTCT |
| YTHDF1-KD#3 | GGAAACGTCCAGCCTAATTCT |
| ANLN-KD#1 | GGTGGTGAAGAGAAATCTTGT |
| ANLN-KD#2 | GCAGATACCATCAGTGATTCT |
| ANLN-KD#3 | GCTACATTCTGTTCCCAAAGG |
| MYBL2-KD#1 | GCCATGGACCAAAGAGGAAGA |
| MYBL2-KD#2 | GGACAGACAATGCTGTGAAGA |
| MYBL2-KD#3 | GCCCAAGAGCACACCTGTTAA |
| AURKA-KD#1 | GGACCTGTTAAGGCTACAGCT |
| AURKA-KD#2 | GGGTCTTGTGTCCTTCAAATT |
| AURKA-KD#3 | GGGCTTTGGAAGACTTTGAAA |
| HMGB2-KD#1 | GGAGGAGGAAGAAGAAGATGA |
| HMGB2-KD#2 | GCGGAATTCTCCAAGAAGTGT |
| HMGB2-KD#3 | GCTAAGCTAAAGGAGAAATAT |
| KIF2C-KD#1 | GCAGTGGCTGAAATACCATTG |
| KIF2C-KD#2 | GGAAATCATGTCTTGTGAAGG |
| KIF2C-KD#3 | GCAAGAATTGGCCAAGAAAGA |
| Control-KD | TTCTCCGAACGTGTCACGT |

KD. knock down

**Table S11. Primers for real-time PCR listed used in this study.**

| Name | Sequence |
|---|---|
| METTL3-F | TTGTCTCCAACCTTCCGTAGT |
| METTL3-R | CCAGATCAGAGAGGTGGTGTAG |
| METTL14-F | GAACACAGAGCTTAAATCCCCA |
| METTL14-R | TGTCAGCTAAACCTACATCCCTG |
| WTAP-F | TTGTAATGCGACTAGCAACCAA |
| WTAP-R | GCTGGGTCTACCATTGTTGATCT |
| KIAA1429-F | TACTTTGAGCCCATTTCTCCTGA |
| KIAA1429-R | GGAATACTGTCTACTGTTCGTCG |
| ALKBH5-F | CGGCGAAGGCTACACTTACG |
| ALKBH5-R | CCACCAGCTTTTGGATCACCA |
| FTO-F | ACTTGGCTCCCTTATCTGACC |
| FTO-R | TGTGCAGTGTGAGAAAGGCTT |
| YTHDF1-F | ATACCTCACCACCTACGGACA |
| YTHDF1-R | GTGCTGATAGATGTTGTTCCCC |
| YTHDF2-F | CCTTAGGTGGAGCCATGATTG |
| YTHDF2-R | TCTGTGCTACCCAACTTCAGT |
| YTHDF3-F | TCAGAGTAACAGCTATCCACCA |
| YTHDF3-R | GGTTGTCAGATATGGCATAGGCT |
| ANLN-F | ATCTTGCTGCAACTATTTGCTCC |
| ANLN-R | TCCTGCTTAACACTGCTGCTA |
| CENPA-F | TTCCTCCCATCAACACAGTCG |
| CENPA-R | CACACCACGAGTGAATTTAACAC |
| KIF2C-F | CTGTTTCCCGGTCTCGCTATC |
| KIF2C-R | AGAAGCTGTAAGAGTTCTGGGT |
| PRR11-F | GACTTCCAAAGCTGTGC TTCC |
| PRR11-R | CTGCATGGGTCCATCC TTTTT |
| DLGAP5-F | AAGTGGGTCGTTATAGACCTGA |
| DLGAP5-R | TGCTCGAACATCACTCTCGTTAT |
| PHF19-F | ACTCGGGACTCCTATGGTGC |
| PHF19-R | CCTCCGTCAGTTTGGACATCA |
| BIRC5-F | AGGACCACCGCATCTCTACAT |
| BIRC5-R | AAGTCTGGCTCGTTCTCAGTG |
| MYBL2-F | GAGGGATAGCAAGTGCAAGGT |
| MYBL2-R | TGCGGTTAGGGAAGTGGCTG |
| CDCA5-F | GACGCCAGAGACTTGGAAATG |
| CDCA5-R | GGACCTCGGTGAGTTTGGAG |
| HMGB2-F | GTGGCCTAGCTCGTCAAGTT |
| HMGB2-R | GCGTACGAGGACATTTTGCC |
| TACC3-F | TCGCCACCAGAAGTTACCG |
| TACC3-R | TCCCGCAGAGGTGTCTGAAA |
| FAM83D-F | GGGAAGGTTCACGAAAAGTTCA |
| FAM83D-R | GACTGGGCATACAGGATTCGG |

| | |
|---|---|
| IQGAP3-F | GCAGCCTATGAACGCCTCA |
| IQGAP3-R | GGAGGGTGCAAAACAGTGG |
| AURKA-F | CAGACTGGATACCGGGACC |
| AURKA-R | CTTCAGCACGTTTTTGCACTG |
| VEGFA-F | AGGGCAGAATCATCACGAAGT |
| VEGFA-R | AGGGTCTCGATTGGATGGCA |
| RANKL-F | CATGTTCGTGGCCCTCCTG |
| RANKL-R | CATGTTCGTGGCCCTCCTG |
| RANK-F | CCGCCTAAGTGGAGATAAGGAAA |
| RANK-R | CGTAGACCACGATGATGTCGC |
| ACTIN-F | TGGCACCCAGCACAATGAA |
| ACTIN-R | CTAAGTCATAGTCCGCCTAGAAGCA |

**Supplementary References**


[1] Yuan JH, Yang F, Wang F. *et al*. A long noncoding RNA activated by TGF-β promotes the invasion-metastasis cascade in hepatocellular carcinoma. Cancer Cell 2014; 25:666-681.

[2] Petroski MD, Deshaies RJ. Function and regulation of cullin-RING ubiquitin ligases. Nat Rev Mol Cell Biol 2005; 6:9-20.

[3] Dominissini D, Moshitch-Moshkovitz S, Salmon-Divon M. *et al*. Transcriptome-wide mapping of N (6)-methyladenosine by m (6)A-seq based on immunocapturing and massively parallel sequencing. Nat Protoc 2013; 8:176-189.

[4] Shen C, Sheng Y, Zhu AC. *et al*. RNA Demethylase ALKBH5 Selectively Promotes Tumorigenesis and Cancer Stem Cell Self-Renewal in Acute Myeloid Leukemia. Cell Stem Cell 2020; 27:64-80. e9.

[5] Li C, Wang S, Xing Z. *et al*. A ROR1-HER3-lncRNA signalling axis modulates the Hippo-YAP pathway to regulate bone metastasis. Nat Cell Biol 2017; 19:106-119.

[6] Sun W, Li SC, Xu L. *et al*. High FLT3 Levels May Predict Sorafenib Benefit in Hepatocellular Carcinoma. Clin Cancer Res. 2020;15;26(16):4302-4312.
